# COLORFUL-Circuit: A Platform for Rapid Multigene Assembly, Delivery, and Expression in Plants

**DOI:** 10.3389/fpls.2016.00246

**Published:** 2016-03-01

**Authors:** Hassan Ghareeb, Sabine Laukamm, Volker Lipka

**Affiliations:** ^1^Department of Plant Cell Biology, Albrecht-von-Haller Institute of Plant Sciences, Georg-August-University of GöttingenGöttingen, Germany; ^2^Department of Plant Biotechnology, National Research CentreCairo, Egypt

**Keywords:** multigene coexpression, circuit design, synthetic biology, binary vectors, gene stacking, plant biotechnology

## Abstract

Advancing basic and applied plant research requires the continuous innovative development of the available technology toolbox. Essential components of this toolbox are methods that simplify the assembly, delivery, and expression of multiple transgenes of interest. To allow simultaneous and directional multigene assembly on the same plant transformation vector, several strategies based on overlapping sequences or restriction enzymes have recently been developed. However, the assembly of homologous and repetitive DNA sequences can be inefficient and the frequent occurrence of target sequences recognized by commonly used restriction enzymes can be a limiting factor. Here, we noted that recognition sites for the restriction enzyme *SfiI* are rarely occurring in plant genomes. This fact was exploited to establish a multigene assembly system called “COLORFUL-Circuit.” To this end, we developed a set of binary vectors which provide a flexible and cost efficient cloning platform. The gene expression cassettes in our system are flanked with unique *SfiI* sites, which allow simultaneous multi-gene cassette assembly in a hosting binary vector. We used COLORFUL-Circuit to transiently and stably express up to four fluorescent organelle markers in addition to a selectable marker and analyzed the impact of assembly design on coexpression efficiency. Finally, we demonstrate the utility of our optimized “COLORFUL-Circuit” system in an exemplary case study, in which we monitored simultaneously the subcellular behavior of multiple organelles in a biotrophic plant–microbe interaction by Confocal Laser Scanning Microscopy.

## Introduction

The coordinated expression of foreign genes in plant cells by genetic engineering is one of the most important technologies in basic and applied plant research (Brophy and Voigt, 2014; Farré et al., 2015). The most common method used for genetic engineering of plants is *Agrobacterium tumefaciens*-mediated transformation. An essential step toward genetic manipulation via *A. tumefaciens* is the construction of binary vectors containing gene expression cassettes. The basic structure of a gene cassette is a promoter, the gene of interest and a transcriptional terminator. For overexpression in plants two promoter elements, the cauliflower mosaic virus CaMV 35S promoter (35S) and the polyubiquitin 10 (UBQ10) promoter of *Arabidopsis thaliana* are commonly used. Both provide constitutive and high levels of gene expression (Grefen et al., 2010). To stop the gene transcription process, the terminators of the cauliflower mosaic virus 35S (T35S), nopaline synthase (Tnos), and octopine synthase (Tocs) of the *A. tumefaciens* have been frequently used (Mitsuhara et al., 1996; Martin et al., 2009). An enormous number of binary plasmids containing combinations of these regulatory elements were developed to enable foreign gene transfer and expression in plant cells (Lee and Gelvin, 2008). The use of these vectors for transgene delivery into plants contributed to our understanding of gene function and genetic networking, and supported the development of plant biotechnology (Dafny-Yelin and Tzfira, 2007). However, their application was mainly limited to expression of one single gene of interest in addition to the selectable marker. Thus, the available molecular toolbox for one-step transformation with more than one transgene is far from being satisfactory despite increasing demands (Atkinson and Urwin, 2012; Ainley et al., 2013; van Erp et al., 2014). Therefore, stable overexpression of multi-transgenes is still considered to be one of the bottlenecks that constrain the engineering of complex metabolic pathways or the improvement of quantitative traits (Bohmert et al., 2002; Naqvi et al., 2010; Giuliano, 2014).

To ease the construction of multigene assemblies, several approaches that make use of DNA overlapping sequences or restriction enzymes have recently been developed (Tzfira et al., 2005; Li and Elledge, 2007; Quan and Tian, 2009; Naqvi et al., 2010; Zeevi et al., 2012; Emami et al., 2013; Liu et al., 2013; Sarrion-Perdigones et al., 2013; Binder et al., 2014; Engler et al., 2014; Hecker et al., 2015). A powerful assembly technique that can be used for joining multiple DNA fragments simultaneously in a single tube is Gibson Assembly (Gibson et al., 2009). Gibson Assembly allows joining the DNA molecules *via* overlapping ends. By concerted action of a 5′ exonuclease, a DNA polymerase and a DNA ligase, single strands of overlapping sequences are generated, annealed, empty gaps filled, and then ligated. Application of Gibson assembly can be limited when homologous sequences and repetitive sequences have to be combined. The same holds true for other overlap-based assembly platforms such as overlap extension polymerase chain reaction, circular polymerase extension cloning, sequence and ligase independent cloning, InFusion® (Clontech) and Multisite Gateway® system (Thermo Fisher Scientific; reviewed in Liu et al., 2013). In addition, these techniques are expensive.

Alternatively, restriction enzyme-based assembly systems can be used for multigene construction. Pioneering work by Tzfira et al. (2005) and Zeevi et al. (2012) provided sets of binary vectors that allow multiple gene cassette assembly using a limited set of DNA-modifying enzymes. In these systems the gene cassettes are ligated together *via* compatible flanking overhangs which are produced by rare-cutting restriction enzymes, zinc-finger nucleases and homing endonucleases. Despite the unquestionable utility and elegance of these systems their applicability is limited due to their dependency on comparatively expensive enzymes. Another powerful approach for simultaneous DNA fragment assembly is provided by Golden Gate and Golden Gate-related systems (Engler et al., 2008; Emami et al., 2013; Sarrion-Perdigones et al., 2013). The latter cloning strategies are based on type II restriction enzymes, such as *BsaI, BsmBI*, and *SapI*, which cut DNA outside of the recognition sites and produce unique overhangs that allow combinatorial DNA assembly. However, we noticed that the restriction enzyme recognition sites used in Golden Gate cloning occur at relatively high frequencies in the genome sequences of plants (see below). Consequently, Golden Gate cloning often requires site-directed mutation of the naturally occurring restriction enzyme recognition sites in the cloned DNA fragment. Therefore this cloning system can be laborious or even of limited utility, in particular when codon optimization is not possible, e.g., in case of promoters or other noncoding DNA sequences. In summary, despite important recent improvements there is still a need to develop DNA assembly methods that can overcome the aforementioned limitations and facilitate multiple gene expression.

Moreover, it has to be kept in mind that the construct design of multigene assemblies may influence the expression efficiency of genetic circuits (Peremarti et al., 2010). For example, repetitive use of the same promoter in multigene constructs may negatively impact on transgene expression (Mette et al., 2000). To assess the performance of individual promoters in multigene assembly and to quantify the gene expression they control, adequate reporter genes are required. Fluorescent proteins (FPs) are instrumental reporters and provide an easy readout to monitor gene expression. Several FPs have been recently developed to cover wide ranges of emission spectra (Kremers et al., 2011). Simultaneous employment of spectrally distant FPs can thus be used to monitor gene expression derived from different gene cassettes (Zeevi et al., 2012; Binder et al., 2014; Hecker et al., 2015). The fluorescent proteins mTurquoise2, Venus, TagRFP-T and mKate2 are spectrally distant, and represent the brightest and most photostable in their spectral ranges (Nagai et al., 2002; Shaner et al., 2008; Shcherbo et al., 2009; Goedhart et al., 2012) making them convenient for multi-reporter purposes.

Besides their suitability to monitor promoter activities and transgene stability, FPs can also be used as markers in plant cell biology. For example, the coexpression of two FP fusions can provide evidence for protein co-localization using conventional fluorescence or confocal microscopy or protein–protein interaction using fluorescence resonance energy transfer (FRET) microscopy (Tzfira et al., 2005; Hecker et al., 2015). More complex live-cell imaging experiments, such as the co-localization of several proteins or studying the spatiotemporal dynamics of several cell organelles, require the use of three or more FPs. However, the FPs-encoding genes generally share a common origin and thus have a high DNA sequence homology (Kremers et al., 2011). This may be problematic as homology of the coding sequences can cause gene silencing (Cogoni and Macino, 1999). Since circuit dynamics can be influenced by the choice of the coding sequence and/or the deployment of the regulatory elements (Peremarti et al., 2010; Brophy and Voigt, 2014), an optimal design of multigene circuit constructs allowing efficient gene expression is necessary.

Here, we developed a set of binary vectors, which we named “COLORFUL-Circuit” that allow for cost-efficient and straightforward cloning of multiple foreign genes, variable FP tagging as well as easy and straightforward promoter exchange. A prominent characteristic of the vectors is that they allow simultaneous assembly of gene cassettes after digestion with one restriction enzyme. We optimized the design of the COLORFUL-Circuit assemblies to increase the efficiency of gene expression of two to four homologous FPs in stable transgenic plants. We believe that the COLORFUL-Circuit platform represents a valuable asset to the genetic circuit design toolbox that provides a number of substantial advantages for scientists working on basic and applied aspects of plant biology.

## Materials and methods

### Plant growth conditions and inoculation with *Golovinomyces orontii*

Seeds of *A. thaliana* (Col-0) or *Nicotiana benthamiana* were vernalized at 4°C for 2 days. Plants were grown in a climate chamber (Johnson Controls, USA) under long day conditions (16/8 h) with 130 μmol·m^−2^·s^−1^ at 22/18 and 25/22°C, respectively. For inoculation experiments with *G. orontii*, 4-week old plants were inoculated with the conidiospores of the fungus and then incubated in a growth chamber under short day conditions (8/16 h) with 130 μmol·m^−2^·s^−1^ at 22/18 and 25/22°C, respectively.

### Frequency determination of restriction enzymes cleavage sites in plant genomes

The chromosomal DNA sequences excluding the randomly assembled nucleotide sequences of *A. thaliana* (ecotype Col-0), rapeseed (*Brassica napus* cultivar Darmor-*bzh*), tomato (*Solanum lycopersicum* cultivar Heinz 1706), and rice (*Oryza sativa* cultivar Nipponbare; Initiative, 2000; Consortium, 2012; Kawahara et al., 2013; Chalhoub et al., 2014) were retrieved from the *Arabidopsis* Information Resource database (ftp://ftp.arabidopsis.org/home/tair/home/tair/Sequences/whole_chromosomes), Genoscope database (http://www.genoscope.cns.fr/brassicanapus/data), Sol genomics network database (ftp://ftp.sgn.cornell.edu/genomes/Solanum_lycopersicum/assembly/current_build), and Rice Genome Annotation Project database (ftp://ftp.plantbiology.msu.edu/pub/data/Eukaryotic_Projects/o_sativa/annotation_dbs/pseudomolecules/version_7.0/all.dir), respectively. Then, the cleavage sites of the restriction enzymes *SfiI, BsmBI, BsaI* and *SapI* in each chromosome were detected and counted using the software Geneious®8.1.7 (Biomatters Ltd). Finally, the number of cleavage sites per megabase (MB) in the entire genome for each restriction enzyme was calculated.

### DNA assembly and plasmid constructions

**Figure 1:**
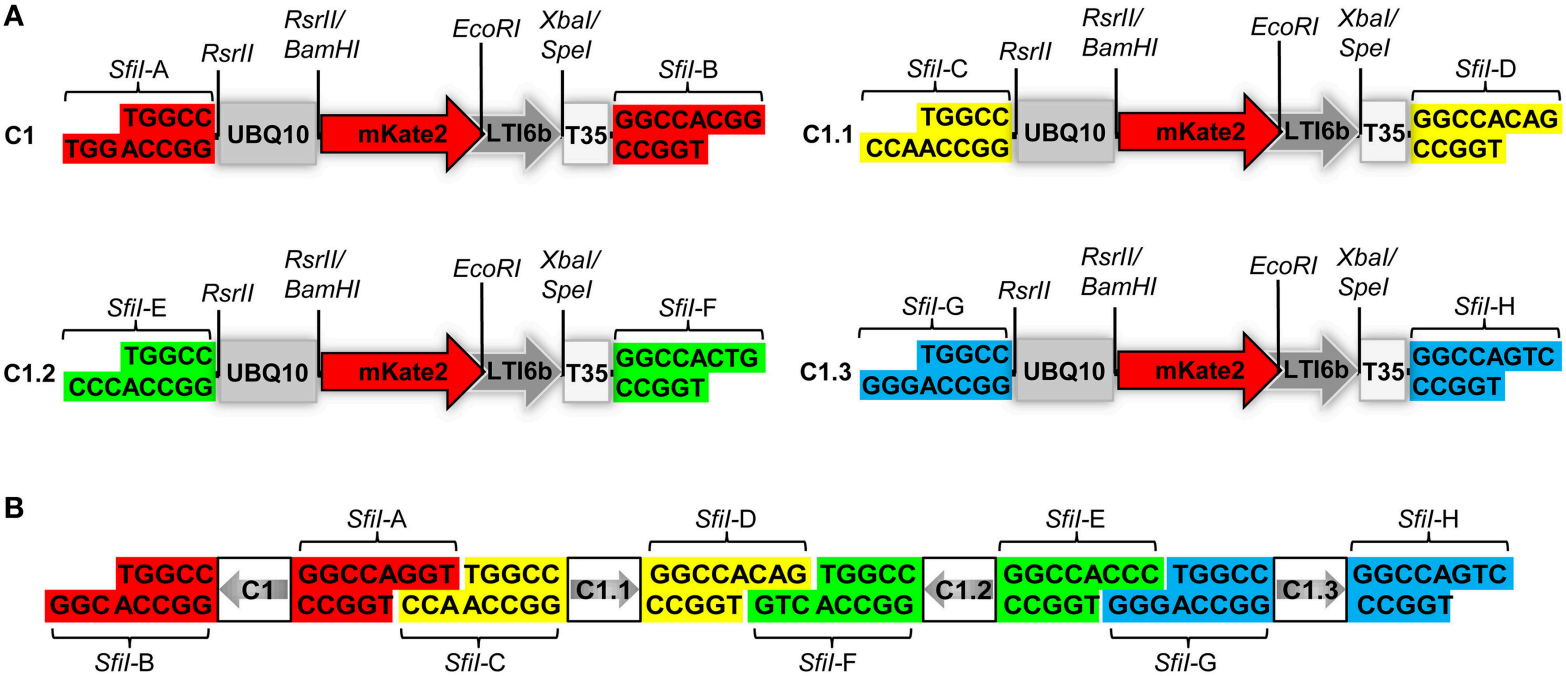
**Structural design of the COLORFUL-Circuit vectors and assembly**. **(A)** Schematic view showing the modular design of the C1 gene expression cassette (1896 bp). The gene cassettes C1, C1.1, C1.2, and C1.3 only differ in the color coded unique *SfiI* overhangs (*SfiI*-A, *SfiI*-B, *SfiI*-C, *SfiI*-D, *SfiI*-E, *SfiI*-F, *SfiI*-G, and *SfiI*-H). UBQ10, polyubiquitin 10 promoter; mKate2, far-red fluorescent protein encoding gene; LTI6b, low temperature induced protein as a membrane marker; T35S, terminator of the cauliflower mosaic virus 35S; *SfiI, RsrII, EcoRI, XbaI/SpeI*, recognition sites of the corresponding restriction enzymes. **(B)** Diagram depicting the concept of COLORFUL-Circuit assembly of four tandemly arranged gene cassettes (C1–C1.1–C1.2–C1.3). The *SfiI* unique overhangs flanking each gene cassette allow combinatorial assembly, after *SfiI* cleavage and subsequent DNA ligation. The gray arrows indicate transcriptional orientations of the gene cassettes. *SfiI* overhangs with the same color flank one gene cassette. Note, that figure elements are not drawn to scale.

For PCR amplification of the DNA fragments that were used for cloning, the oligonucleotide primers listed in Supplementary Table S1 were used. The basic gene cassette (C1) modules were cloned in a pUC19 cloning vector (Clontech, Saint-Germain-en-Laye, France), which was modified to contain the *SfiI*-A and *RsrII, SfiI*-B recognition sites. The UBQ10 was cloned via *RsrII* cloning sites, and mKate2 was fused to UBQ10 using *BamHI* restriction sites, which were present in the reverse primer that was used to amplify the UBQ10 and the forward primer used for generation of the mKate2 fragment. Likewise, we sequentially fused LTI6b and T35S using the cloning sites *EcoRI*/*SpeI*, and *SpeI*/*SfiI*-B, respectively, to generate the C1 cassette. The C1.1, C1.2, and C1.3 cassettes were generated by PCR amplification of the C1 cassette using pairs of oligonucleotide primers that replace the *SfiI*-A and *SfiI*-B with *SfiI*-C and *SfiI*-D, *SfiI*-E and *SfiI*-F, and *SfiI*-G and *SfiI*-H, respectively as indicated in Figure 1A. Meanwhile, four binary vector backbones were PCR-amplified from the plasmid pXNS2pat-YFP (accession number KF499077; Dahncke and Witte, 2013) using four pairs of primers that add distinct *SfiI* cleavage sites in the flanks of the backbones to specifically allow ligation with the overhangs of either C1, C1.1, C1.2, or C1.3 gene cassette as indicated in Supplementary Figure S1. The gene cassettes C1, C1.1, C1.2, or C1.3 and their corresponding vector backbones were cleaved with *SfiI* (NEB, Germany), separated on agarose gel electrophoresis, and then cleaned up from the gel using innuPREP DOUBLEpure Kit (Analytik Jena, Germany). For the *SfiI* cleavage, 0.5–1 μg DNA, 2 μl CutSmart® buffer and 0.2–0.5 μl *SfiI* (20 unit/μl) were added in 20 μl total volume, and then incubated for 1–16 h at 50°C in a thermocycler (MyCycler™, Bio-Rad, USA). Note that *SfiI* functions more efficiently in excess of DNA containing multiple cleavage sites of the enzyme. The *SfiI*-cleaved vector backbone was then ligated with the corresponding gene cassette to produce pC1, pC1.1, pC1.2, and pC1.3. To generate binary vectors expressing the fluorescent organelle markers, the gene encoding TagRFP-T-SKL was PCR amplified and cloned into pC1.1 *via BamHI* and *SpeI* restriction sites to produce pC2. Venus and mTurquoise2 were cloned into pC1.2 and pC1.3, respectively, via *BamHI* and *EcoRI*. Then pC1.2 and pC1.3 were cleaved with *EcoRI* and *SpeI*, and then MAP4 and N7, which were PCR amplified using primers that add 20 bp overlapping to 3′-ends of Venus or mTurquoise2, and 5′-ends of T35S, were fused into the vectors via Gibson Assembly (NEB, Gibson et al., 2009) to generate pC3 and pC4, respectively. The UBQ10 promoters in pC2 and pC4 were replaced with the 35S promoters using the *RsrII* cloning sites. The Tocs terminator was PCR amplified and replaced the T35S of pC2 using the *SpeI* and *SfiI* cloning sites.

To construct multigene binary vectors, we amplified the backbone of pGreenII “hosting vectors” (Hellens et al., 2000) by PCR using primer pairs, which add *SfiI* recognition sites compatible with the free *SfiI* overhangs of the gene cassettes to be assembled. The vector backbones and the plasmids pC1, pC2, pC3, and pC4 were digested with *SfiI*, separated on agarose gel electrophoresis, and then the corresponding DNA fragments were cleaned up from the gel. The gene cassettes C2 and C3 were ligated with a hosting vector, which harbors compatible overhangs to the *SfiI*-C and *SfiI*-E sites, to produce the plasmid containing double-gene cassette pC2–C3. Likewise, we produced the plasmid containing triple-gene cassettes pC1–C3 and quadruple-gene cassettes pC1–C4 (version I). For assembling the multigene into a hosting binary vector, we ligated 50 ng vector backbone in 1:1 molar ratio with the *SfiI*-cleaved gene cassettes in a 20 μl total reaction that contained 2 μl buffer and 1 μl T4 DNA ligase (1 unit/μl; Thermo Fisher Scientific, Germany). The gene cassettes C2i and C3i were generated by PCR amplification from pC2 and pC3 using primer pairs, which contained swapped recognition sites of *SfiI*-C and *SfiI*-D and *SfiI*-E and *SfiI*-F, respectively. The C2i and C3i cassettes were *SfiI* cleaved and then ligated with the *SfiI*-cleaved vector backbones of pC2 and pC3 to produce the plasmids pC2 and pC3i, respectively. The plasmid pC1–C4 (version II) was generated by ligating the *SfiI*-cleaved cassettes C1, C2i, C3i, and C4, and the quadruple-gene hosting vector backbone. Similarly, the pC1–C4 (version III) and pC1–C4 (version IV) were constructed except that the C3i was exchanged with C3 in the earlier and C2 was used instead of C2i in the later.

### Transient plant transformation

A single colony of *A. tumefaciens* was used to inoculate 2 ml LB medium with the appropriate antibiotics and was grown for 1–2 days at 28°C. Subsequently, 0.5 ml of the grown culture were used to inoculate 4.5 ml of LB medium containing the appropriate antibiotics and 20 μM sterile acetosyringone. Cultures were grown overnight at 28°C with shaking and then were centrifuged for 10 min at 4500 × g. The pellet was resuspended in an infiltration solution containing 10 mM MgCl_2_, 10 mM MES-K (pH 5.6) and 100 μM acetosyringone to obtain a final OD_600_ of 0.4. The cultures were incubated at room temperature for 2–4 h. Then, the abaxial side of *N. benthamiana* leaves was infiltrated with the bacterial suspension. After 2–3 days, the fluorescence signals were detected using Confocal Laser Scanning Microscopy (CLSM).

### Stable plant transformation

A double floral dip protocol was combined from previously established protocols (Clough and Bent, 1998; Davis et al., 2009). First, six pots, 8 cm^2^, each containing five plants were used for the transformation. As the first inflorescence shoots were emerged, they were excised and 1 week later the first floral dip was preceded. Three days before the transformation, *Agrobacterium* cultures were inoculated in 25 ml LB liquid medium containing 50 μg/ml Rifampicin, 30 μg/ml Gentamicin, 2.5 μg/ml Tetracycline, and 50 μg/ml Kanamycin. The cultures were incubated at 28°C with shaking (190 rpm). Two days later, the grown cultures were inoculated in 500 ml of LB liquid medium with the same antibiotic concentrations and incubated for 24 h at 28°C with shaking (190 rpm). The bacterial cultures were centrifuged at 4500 × g for 20 min at room temperature. The pellet was resuspended in 500 ml infiltration medium (0.5X MS with Gamborg B5 vitamins, 5% sucrose, and 150 μl/l Silwet L-77). The suspension was transferred into a 500-ml beaker, where the plants were dipped. Afterwards, the plants were placed into a transparent plastic bag and incubated on their side in the dark for 16–24 h. In the next day, the plants were returned back to normal growth conditions. A second floral dip was performed 5 days later using bacteria grown in YEBS medium with half concentrations of the antibiotics as the main culture. The bacteria were resuspended in infiltration medium with 300 μl/l Silwet L-77. We obtained a high transformation rates using this protocol. After 4 weeks, seeds were collected. Stably transformants were selected by on-soil Basta® selection. One-week-old seedlings were sprayed with 1:1000 Basta (containing 120 mg\ml phosphinothricin, Bayer CropScience, Germany). The treatment was conducted every 2 days and repeated three times. The stable transformation rate was determined as the percent of Basta-resistant T1 plants from the total treated plants. Survived plants, which were 2–3 weeks old, were used for CLSM.

### Transgene stability assay

Transgenic plants that carry single T-DNA insertion for C1–C4 (III) or C1–C4(IV) were used to study the transgenerational stability. For this purpose, seeds of the T2 generations from three independent lines for each construct were sterilized by adding 500 μl of 70% ethanol and mixing using a Rotator (20 rpm) for 5 min. The 70% ethanol was replaced by 500 μl of 99% ethanol for 1 min and then discarded. The seeds were air-dried, then sown onto ½MS/MES agar medium (MS 2.2 g/l, MES 0.5 g/l, plant agar 7 g/l, pH 5.8) supplemented with 10 mg/l PPT and kept at 4°C for 2 days. The seedlings were grown in a climate chamber (Johnson Controls, United Stated of America) under short day conditions (8 h light/16 h dark) with 150 μmol·m^−2^·s^−1^ at 22/18°C for 12 days. The Basta-resistant plants were screened for expression of the individual organelle markers using CLSM.

### Confocal laser scanning microscopy

Leaves from transiently transformed *N. benthamiana* or T1 or T2 of stably transformed *Arabidopsis* were used for CLSM. All images were captured using HyD and PMT detectors and 20x or 63x objectives of TSC-SP5 microscope (Leica, Bensheim, Germany). mTurquoise2 and Venus were excited using 458 and 514 nm lines of an argon laser, respectively, whereas TagRFP-T and mKate2 were excited with 561 and 594 nm lasers. Fluorescence emissions were detected at 462–485 nm for mTurquoise2, 520–540 nm for Venus, 565–580 nm for TagRFP-T, and 620–640 nm for mKate2. The images were sequentially scanned with a resolution of 512 × 512 pixels and 200 Hz scanning speed. A linear spectral unmixing was performed for the TagRFP-T and mKate2 channels. The maximum projection and image merging and linear spectral unmixing were performed using imageJ.

## Results and discussion

### Principle and basic vectors of the COLORFUL-circuit platform

Expression of foreign genes *in planta* via *Agrobacterium*-mediated transformation requires construction of binary vectors. We generated a set of binary vectors to allow single gene or multigene construction, delivery and expression in plants. For single gene expression, we started by generating a modular gene cassette (C1) that allows simple cloning steps and contains easily exchangeable modules: a UBQ10 promoter (Grefen et al., 2010), a gene encoding the far-red fluorescent reporter gene mKate2 (Shcherbo et al., 2009) fused to the plasma membrane protein low temperature induced protein 6b (LTI6b; Cutler et al., 2000), followed by a T35S terminator (Mitsuhara et al., 1996; Figure 1A). For generation of C1, we first introduced *RsrII* recognition sites at the left and right flanks of UBQ10 by PCR site-directed mutagenesis (Figure 1A). The restriction enzyme *RsrII* recognizes the sequence CG^∧^GWCCG, allowing us to produce two distinct recognition sites by assigning two different nucleotides at position four of the recognition sites at the left (adenine) and right border (thymine). Consequently, upon *RsrII* cleavage two unique three-nucleotide overhangs are produced that allow a subsequent unidirectional exchange of promoter sequences. Next, we fused the mKate2 reporter gene to the 3′-end of the UBQ10 promoter using *BamHI* restriction sites present in the 3′-oligonucleotide primer used for generation of the UBQ10 module and the 5′-oligonucleotide primer used for PCR-amplification of the mKate2 module (Supplementary Table S1). Likewise, in subsequent steps we fused LTI6b via *EcoRI*/*SpeI* and finally, T35S via *SpeI*/*SfiI* which produced the full-length gene cassette C1 depicted in Figure 1A.

Notably, the 5′- and 3′-ends of the C1 cassette contain recognition sites for the restriction enzyme *SfiI*, namely *SfiI*-A and *SfiI*-B, which stem from the cloning vector (Figure 1A). *SfiI* is a type II restriction enzyme that recognizes the sequence GGCCNNNN^∧^NGGCC. The nucleotides at position 6–8 of the *SfiI* recognition site typically define a sticky overhang. These overhang sequences can be freely modified to generate unique non-palindromic ends allowing unidirectional cloning. We exploited this fact to generate additional gene cassettes that can be later utilized for multigene assembly. To this end, we PCR-amplified the intact C1 cassette with oligonucleotide primers, which exchange the *SfiI*-A and *SfiI*-B sites with two other distinct *SfiI* cloning sites *SfiI*-C and *SfiI*-D, thus generating a derivative cassette called C1.1 (Figure 1A; Supplementary Table S1). Similarly, we generated two other gene cassettes C1.2 and C1.3, in which the unique *SfiI* recognition sites *SfiI*-E and *SfiI*-F, and *SfiI*-G and *SfiI*-H, respectively, replaced the *SfiI*-A and *SfiI*-B sites (Figure 1A). Each unique *SfiI* overhang was designed to complement with one other *SfiI* overhang in a manner that allows the assembly of four gene cassettes in alternate orientations (Figure 1B). We exploited these gene cassettes below for the establishment of a platform, which we named “COLORFUL-Circuit,” for multigene assembly, delivery, and expression of up to four different genes of interest controlled by distinct promoters and terminators.

### Establishment of multicolor fluorescent organelle markers for *Agrobacterium*-mediated transformation of plants

**Figure 2:**
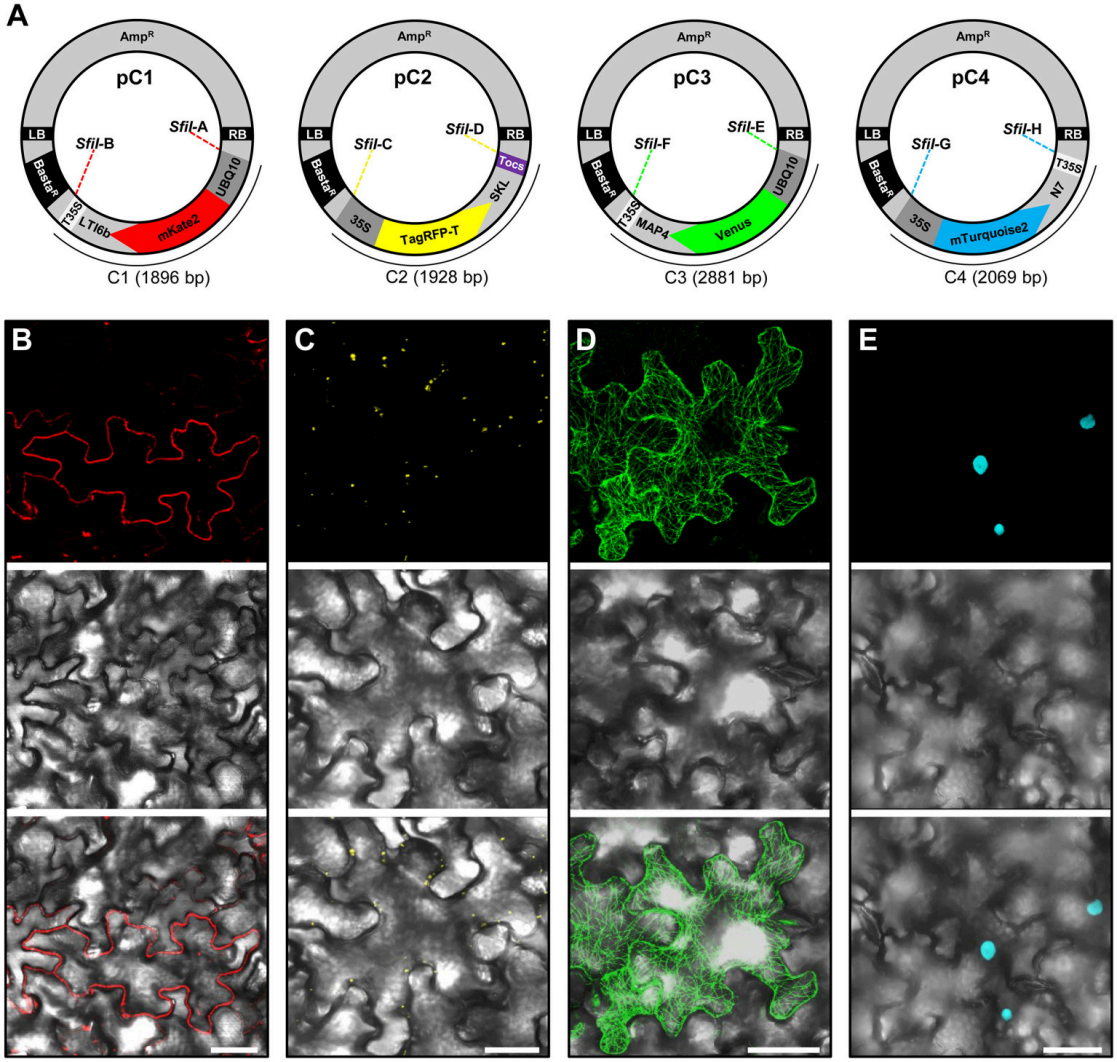
**Construction and transient expression of fluorescent organelle markers**. **(A)** Schematic view of the modular pC1 (6178 bp), pC2 (6210 bp), pC3 (7163 bp), and pC4 (6351 bp) binary vectors expressing fluorescent organelle markers. The vector backbone (4282 bp) is represented outside of the *SfII* recongintion sequences (*SfiI*A, *SfiI*B, *SfiI*C, *SfiI*D, *SfiI*E, *SfiI*F, *SfiI*G, and *SfiI*H) that flank the gene cassettes encoding the fluorescent organelle markers C1 (1896 bp), C2 (1928 bp), C3 (2881 bp), and C4 (2069 bp). The vector backbone was amplified from the plasmid pXNS2pat-YFP. UBQ10, polyubiquitin 10 promoter; 35S, cauliflower mosaic virus CaMV 35S promoter; T35, terminator of the cauliflower mosaic virus 35S; Tocs, terminator of octopine synthase; mKate2, TagRFP-T, Venus and mTurquoise2, fluorescent protein encoding genes; LTI6b, low temperature induced protein as a membrane marker; SKL, peroxisomal targeting sequence; MAP4, microtubule binding domain of the mouse microtubule-associated protein 4; N7, nuclear localization signal; Basta^R^, selectable marker conferring Basta resistance in plants; Amp^R^, selectable marker conferring ampicillin resistance in *E. coli* and *A. tumefaciens*; LB/RB, left/right borders of T-DNA. The figure is not drawn to scale. **(B–E)** *Agrobacterium*-mediated transient expression of the fluorescent organelle markers indicated in **(A)**. **(B)** Membrane localization of mKate2-LTI6b produced from pC1. **(C)** Peroxisomal localization of TagRFP-T-SKL produced from pC2. **(D)** Microtubule localization of Venus-MAP4 produced from pC3. **(E)** Nuclear localization of mTurquoise2-N7 produced from pC4. Top, middle and bottom panels in **(B–E)** represent fluorescence, bright field and overlay of confocal images. The images in **(C–E)** represent maximum projections of z-stacks obtained by confocal laser scanning microscopy. Scale bar = 50 μm.

To allow *Agrobacterium*-mediated transformation of the gene cassettes, we cloned the C1, C1.1, C1.2, and C1.3 cassettes separately into binary vectors. This was accomplished by PCR amplification of the vector backbone of pXNS2pat-YFP (Dahncke and Witte, 2013) using oligonucleotide primers that add terminal *SfiI* cloning sites, which permitted subsequent ligation with the *SfiI* overhangs (*SfiI*-A and *SfiI*-B) of the C1 cassette to produce the binary vector pC1 (Figure 2A, Supplementary Figure S1). Similarly, we generated the C1.1-, C1.2- and, C1.3-containing binary vectors pC1.1, pC1.2, and pC1.3, respectively (Supplementary Figure S1).

To allow monitoring of synchronous gene expression in later multigene assembly vectors, we first cloned into our basic vectors pC1.1, pC1.2, and pC1.3 different gene fusions encoding fluorescent organelle markers and regulatory elements. To this end, the peroxisomal targeting sequence SKL (Keller et al., 1991), the microtubule-binding domain of the mouse microtubule-associated protein 4 (MAP4; Aizawa et al., 1991; West et al., 1991), and the nuclear localization signal N7 (Cutler et al., 2000), were used to generate the fluorescent organelle markers TagRFP-T-SKL, Venus-MAP4 and mTurquoise2-N7 (Figure 2A). This was achieved by introducing the cloning sites *BamHI* and *SpeI* into TagRFP-T-SKL and subsequent replacement of mKate2-LTI6b in pC1.1 to produce the vector pC2 (Figure 2A). Similarly, mKate2 in pC1.2 and pC1.3 was replaced with Venus and mTurquoise2, respectively, using the *BamHI* and *EcoRI* cloning sites. Subsequently, pC1.2 and pC1.3 were cleaved with *EcoRI* and *SpeI*, and MAP4 and N7 were fused to 3′-ends of Venus and mTurquoise2, and 5′-ends of T35S using Gibson Assembly (see Materials and Methods, Gibson et al., 2009) to generate the binary vectors pC3 and pC4, respectively (Figure 2A). Finally, the promoters of the gene cassettes C2 and C4 were exchanged with the 35S promoter using *RsrII*, and the terminator of pC2 was exchanged with Tocs using *SpeI*/*SfiI* (Figure 2A). To test the functionality of the individual organelle marker plasmids pC1, pC2, pC3, and pC4, we transiently expressed them separately in *N. benthamiana* leaves *via Agrobacterium*-mediated transformation and subsequently performed CLSM. These analyses showed that the four organelle markers were expressed and localized to the respective organelles as expected (Figures 2B–E).

### COLORFUL-circuit simplifies multigene assembly

As outlined above, gene cassettes harbored by the vectors pC1–pC4 are characterized by unique overhangs that are produced by a single enzyme, *SfiI*, and allow simultaneous multigene assembly (Figure 1B). Another notable feature of *SfiI* is that cleavage sites for this enzyme rarely occur in the genomes of *A. thaliana*, rapeseed, tomato and rice with only 3.1–40.8 sites per MB on average (Table 1 and Supplementary Tables S2–S5). This provides a clear advantage of *SfiI* over the enzymes *BsaI, BsmBI*, and *SapI*, which are commonly used for Golden Gate and Golden Gate-related cloning systems (Emami et al., 2013; Sarrion-Perdigones et al., 2013; Binder et al., 2014; Engler et al., 2014), as recognition sequences for these enzymes occur at 216.7–267.2, 98–341.9, and 69.5–150.6 sites per MB, respectively (Table 1 and Supplementary Tables S2–S5). Consequently, the *SfiI* cleavage sites in our vectors allow an easier and straightforward assembly of large genomic DNA fragments. In addition, *SfiI* is a relatively cheap restriction enzyme, offering a cost-efficient and simple alternative to other DNA assembly systems.

**Table 1:** **Cleavage site frequency of the restriction enzymes *SfiI, BsmBI, BsaI*, and *SapI* occurring in the genomes of *A. thaliana*, rapeseed, tomato and rice**.

**Name of organism**	Size (MB^*^)	Number of cleavage sites per MB^*^			
		***SfiI***	***BsaI***	***BsmBI***	***SapI***
*A. thaliana*	119.146348	3.1	265.9	275.8	150.6
Rapeseed	650.398471^#^	4.9	237.7	257.3	115.9
Tomato	703.594776	4.5	216.7	98	69.5
Rice	373.245519	40.8	267.2	341.9	134.2

* *Megabase*,

# *genome size without the randomly assembled nucleotide sequences*.

**Figure 3:**
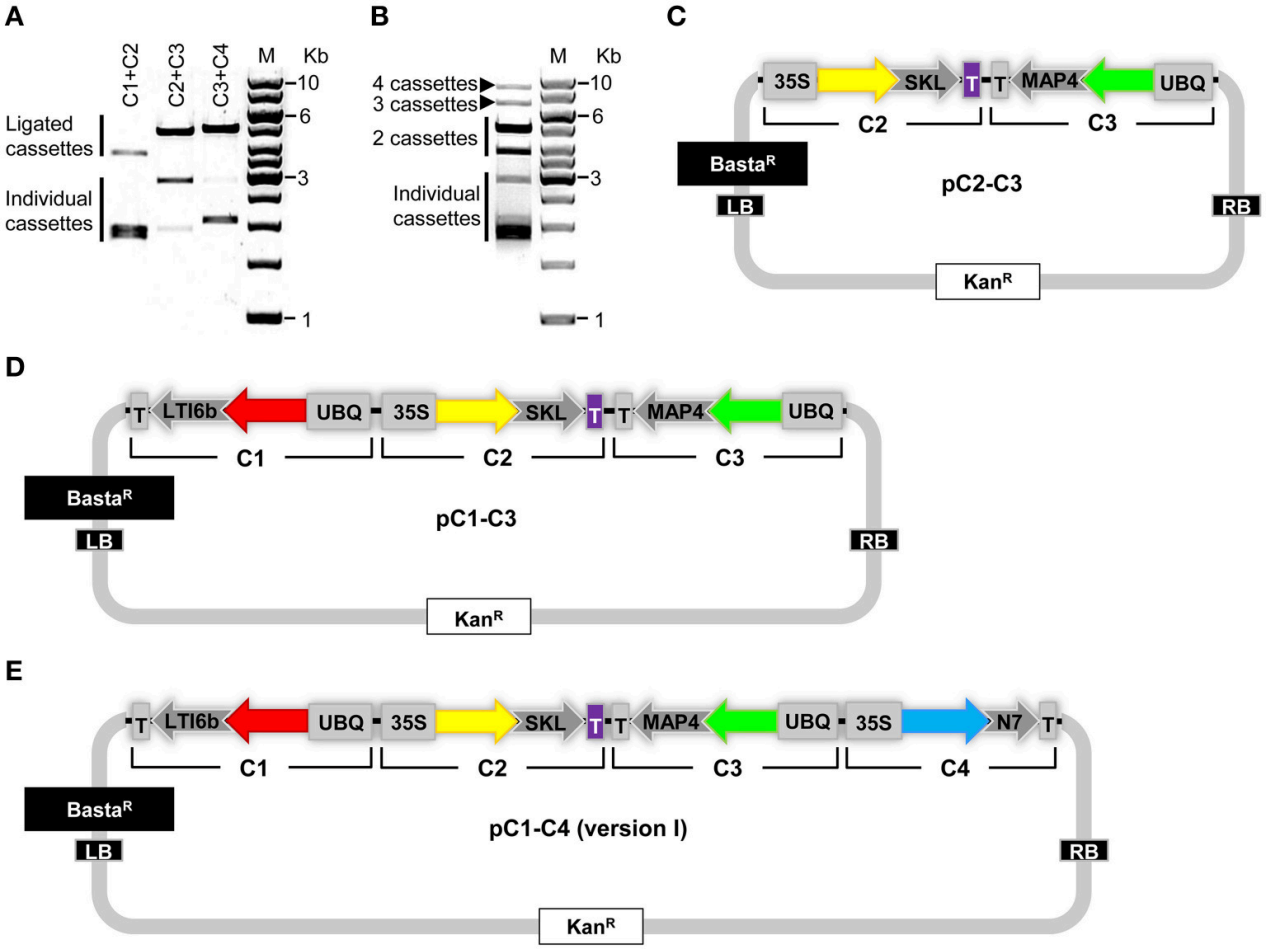
**Generation of COLORFUL-Circuit multigene assemblies**. **(A)** Ligation of the *SfiI*-cleaved gene cassettes C1, C2, C3, and C4 followed by agarose gel electrophoresis verified the production of higher molecular weight DNA fragments corresponding to the assembled molecules. C1, 1896 bp, and C2, 1928 bp (ligated C1 + C2 = 3824 bp); C2 and C3, 2881 bp (ligated C2 + C3 = 4809 bp); and C3 and C4, 2069 bp (ligated C3 + C4 = 4950 bp). M: GeneRuler™ 1-Kb DNA ladder. **(B)** DNA assembly of the four individual gene cassettes (C1, C2, C3, and C4) upon ligation. All possible unidirectionally ligated gene cassettes are produced; (C1 + C2 = 3824 bp; C2 + C3 = 4809 bp; C3 + C4 = 4950 bp), (C1 + C2 + C3 = 6705 bp; C2 + C3 + C4 = 6878 bp), and (C1+ C2 + C3 + C4 = 8774 bp). M: GeneRuler™ 1-Kb DNA ladder. **(C–E)** Modules of the COLORFUL-Circuit multigene binary vectors that allow double-gene cassette assembly (pC2–C3), Triple-gene cassette assembly (pC1–C3) and quadruple-gene cassette assembly [pC1–C4 (version I)], respectively. C1, C2, C3, and C4: individual gene cassettes. The vector backbone was amplified from the plasmid pGreenII. UBQ: polyubiquitin 10 promoter; 35S: cauliflower mosaic virus CaMV 35S promoter; T: terminator of the cauliflower mosaic virus 35S (gray) and terminator of octopine synthase (purple); red, yellow, green and turquoise arrows: fluorescent protein encoding genes of mKate2, TagRFP-T, Venus and mTurquoise2, respectively; LTI6b, low temperature induced protein as a membrane marker; SKL, peroxisomes targeting sequence; MAP4, microtubule binding domain of the mouse microtubule-associated protein 4; N7, nuclear localization signal; Basta^R^, selectable marker confers Basta resistance in plants; Kan^R^, selectable marker confers kanamycin resistance in *E. coli* and *A. tumefaciens*; LB/RB, left/right borders of T-DNA. The figures are not drawn to scale.

Next, we tested the practicability of our COLORFUL-Circuit multigene assembly system. For this purpose, we first digested pC1, pC2, pC3, and pC4 with *SfiI*, separated the gene cassettes from the plasmid backbones using agarose gel electrophoresis, and then extracted the corresponding gene cassettes C1 (1896 bp), C2 (1928 bp), C3 (2881 bp), and C4 (2069 bp) from the agarose gel. Subsequently, the three adjacent gene cassette pairs (C1 + C2, C2 + C3, and C3 + C4) were separately ligated and the ligation reactions were checked by agarose gel electrophoresis (Figure 3A). These experiments confirmed the ligation of the gene cassettes as the expected sizes of each ligated pair was evident (C1 + C2 = 3824 bp; C2 + C3 = 4809 bp; C3 + C4 = 4950 bp; Figure 3A). In a next step, we tested whether all four gene cassettes can be ligated in a single cloning step. Again, agarose gel electrophoresis confirmed the production of all possible assemblies as we were able to detect ligated DNA fragments corresponding to double (C1 + C2 = 3824 bp; C2 + C3 = 4809 bp; C3 + C4 = 4950 bp), triple (C1 + C2 + C3 = 6705 bp; C2 + C3 + C4 = 6878 bp), and quadruple (C1+ C2 + C3 + C4 = 8774 bp) gene cassette assemblies that were unidirectionally ligated (Figure 3B).

Finally, we generated binary vectors hosting the multigene assemblies for *Agrobacterium*-mediated plant transformation. To this end, we introduced *SfiI* restriction sites matching the 5′- and 3′-overhangs of our gene cassettes C1 to C4 into the vector backbone of pGreenII (Hellens et al., 2000). Then, we produced vectors containing double (pC2–C3; Figure 3C), triple (pC1–C3; Figure 3D) and quadruple [pC1–C4 (version I), Figure 3E] gene cassettes using *SfiI* restriction and subsequent ligation. It is important to notice that the multigene hosting vectors contain selectable markers that confer kanamycin resistance in *Escherichia coli* and *A. tumefaciens* (Figures 3C–E). Since the single-gene cassette-containing plasmids pC1, pC2, pC3, and pC4 confer bacterial resistance to ampicillin (Figure 2A), counter-selection for kanamycin avoids contamination with traces of the individual undigested plasmids and thereby maximizes the rate of correct recombinants. In support of this, we typically obtained vectors harboring the correct multigene assemblies at an average frequency of 87% (Supplementary Figure S2). Taken together, these experiments confirmed that our COLORFUL-Circuit system can be efficiently used to generate multigene assemblies.

### Colorful-circuit assemblies allow robust transient coexpression of up to four genes of interest

Transient coexpression of multiple genes is conventionally achieved by co-transformation of single plasmids *via* agroinfiltration or gene bombardment. This strategy is laborious and time consuming. Moreover, uniform coexpression of transgenes in all transformed cells cannot be obtained. For example, co-transformation of vectors encoding two genes from separate T-DNAs was shown to yield 74–84% of cells that coexpressed both genes, whereas up to 100% of cells coexpressed both genes when encoded from a single plasmid (Hecker et al., 2015). To analyze coexpression efficiency in our double, triple and quadruple gene assemblies, we first transiently expressed the corresponding COLORFUL-Circuit constructs pC2–C3, pC1–C3, and pC1–C4 (version I) in *N. benthamiana* leaves *via* agroinfiltration and monitored the expression of fluorescent organelle markers by CLSM. These analyses showed that all organelle markers encoded by the respective constructs were efficiently coexpressed (Supplementary Figure S3A) and showed the expected subcellular localization (Supplementary Figure S3B). With a maximum number of up to four efficiently coexpressed transgenes our system provides an advantage over the recently published “Binary 2in1 vector”-system, which allows coexpression of up to two genes from the same T-DNA (Hecker et al., 2015).

### Stable transformation and coexpression rates negatively correlate with the number of genes within the assembly

**Figure 4:**
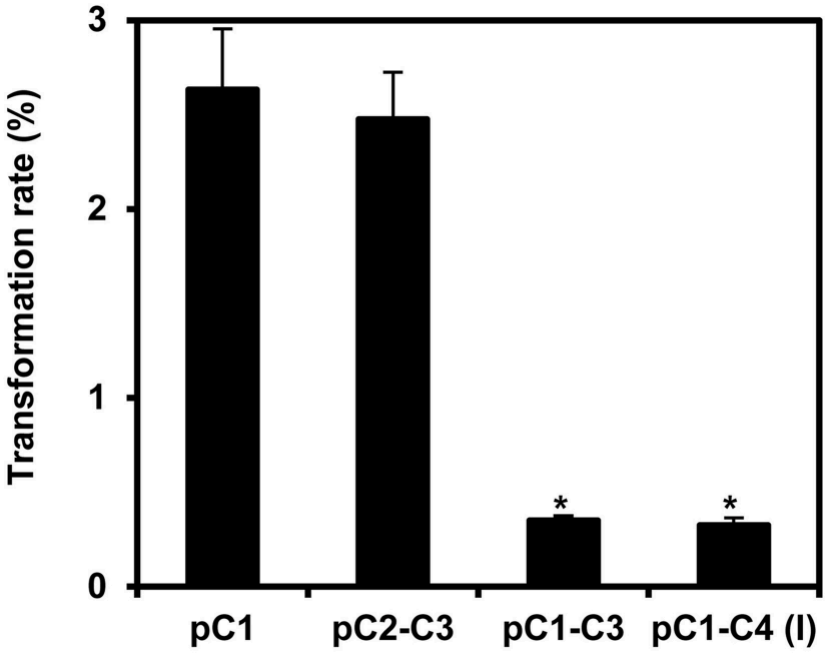
**Transformation rates of the COLORFUL-Circuit T-DNAs stably integrated in *Arabidopsis***. Transformation rate was calculated as percent of Basta-resistant T1 plants obtained from seeds that were harvested from plants transformed with *Agrobacterium* strains harboring binary vectors containing either single-(pC1), double-(pC2–C3), triple-(pC1–C3), or quadruple-gene cassettes [pC1–C4 (version I)] in addition to the Basta selectable marker. The data represent the means ± SEM of three experiments. Asterisks indicate significant differences in comparison to pC1 using student *t*-test, *p* < 0.01.

Next, we stably transformed *Arabidopsis* Col-0 plants with our COLORFUL-Circuit constructs, determined the transformation rates and analyzed the expression of the individual reporter genes encoded by the respective assemblies. With transformation rates of 2.6 and 2.5%, respectively, the single gene cassette C1 and the double-gene cassette C2–C3 were efficiently delivered and integrated into the plant genomes (Figure 4). In contrast, the transformation rates determined for the triple construct C1–C3 and the quadruple construct C1–C4, were 7.5- and 8-fold lower, respectively, compared to the transformation rate observed for C1 (Figure 4). Thus, our data support the previous observation that transformation rates of potato and tobacco plants negatively correlate with the size of the T-DNA that is to be delivered (Bohmert et al., 2002). However, with transformation rates between 0.32 and 2.6% we were still able to isolate a satisfactory number of transformants for all constructs, which were used for further analysis.

**Figure 5:**
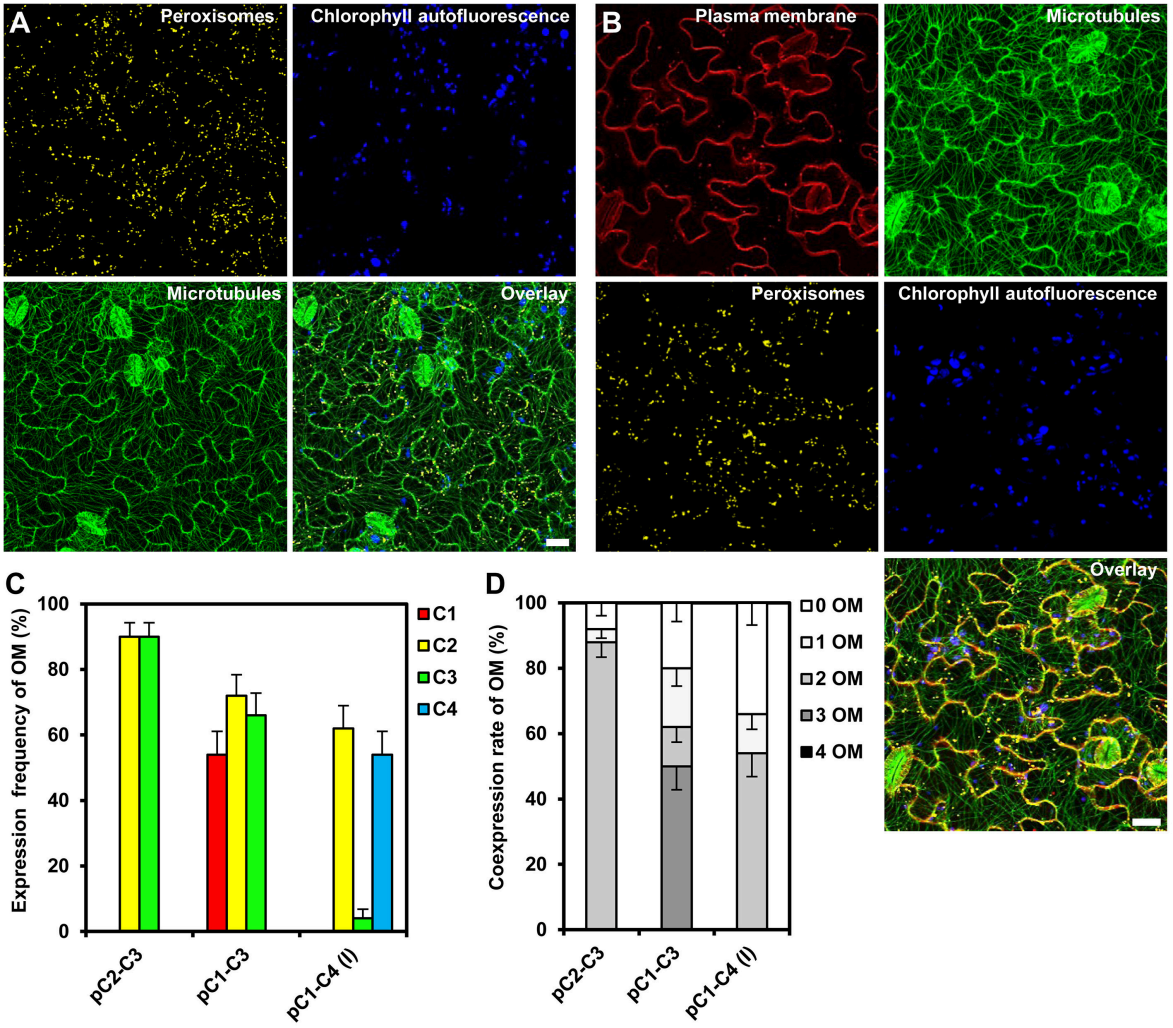
**Gene expression efficiency of the COLORFUL-Circuit assemblies in stable *Arabidopsis* transformants**. **(A)** and **(B)** Leaf cells of transgenic *Arabidopsis* plants stably expressing double and triple organelle markers encoded by C2–C3 and C1–C3 T-DNAs, which are depicted in Figures 3C,D, respectively. Chlorophyll autofluorescence is provided as a control. The images are maximum projections of z-stacks obtained by CLSM. Scale bar = 20 μm. **(C)** Expression frequency of organelle markers (OM) calculated as percent of T1 transgenic plants expressing C1 (plasma membrane marker), C2 (peroxisomal marker), C3 (microtubule marker), or C4 (nuclear marker). Data represent percentages ± SEM (*n* = 50 plants). **(D)** Coexpression rate of organelle markers (OM) calculated as percent of T1 transgenic plants that coexpress the indicated number of organelle markers. *n* = 50 plants.

To analyze the expression of the individual reporter genes in the obtained transgenic *Arabidopsis*, we selected 50 stable cassette transformants per assembly and detected the fluorescent gene products by CLSM. We were able to identify individual transgenic plants expressing all two or three organelle markers encoded by the respective double and triple gene assemblies C2–C3 and C1–C3, respectively (Figures 5A,B). In order to obtain quantitative data, we determined both, the expression frequency of every individual organelle marker and, in addition, the coexpression rates of all reporter genes encoded by the respective multigene construct in each individual transgenic plant. These analyses showed that either organelle marker encoded from the double-gene assembly C2–C3 was detectable in 90% of the transformants (Figure 5C), and that both organelle markers were coexpressed in 88% of the plants (Figure 5D). Thus, compared to the 10% of all *Arabidopsis* transformants that coexpressed two genes using the Binary 2in1 vector system (Hecker et al., 2015), our COLORFUL-Circuit pC2–C3 provided higher rates of coexpression (Figure 5D). However, in plants carrying the triple reporter C1–C3 assembly, the expression frequency of the individual organelle markers was reduced to 54–72% (Figure 5C), with only 50% of the analyzed plants coexpressing all three organelle markers (Figure 5D). Finally, and in marked contrast to the fact that we were able to robustly detect all four fluorescent organelle markers in our transient expression experiments (Supplementary Figures S3A,B), none of the 50 selected transgenic plants transformed with the quadruple C1–C4 assembly coexpressed all four reporter genes (Figures 5C,D). Together, our data show that not only the transformation rates, but also the coexpression rates negatively correlate with the number of genes encoded within the assembly.

### Assembly design impacts efficiency of multigene coexpression

**Figure 6:**
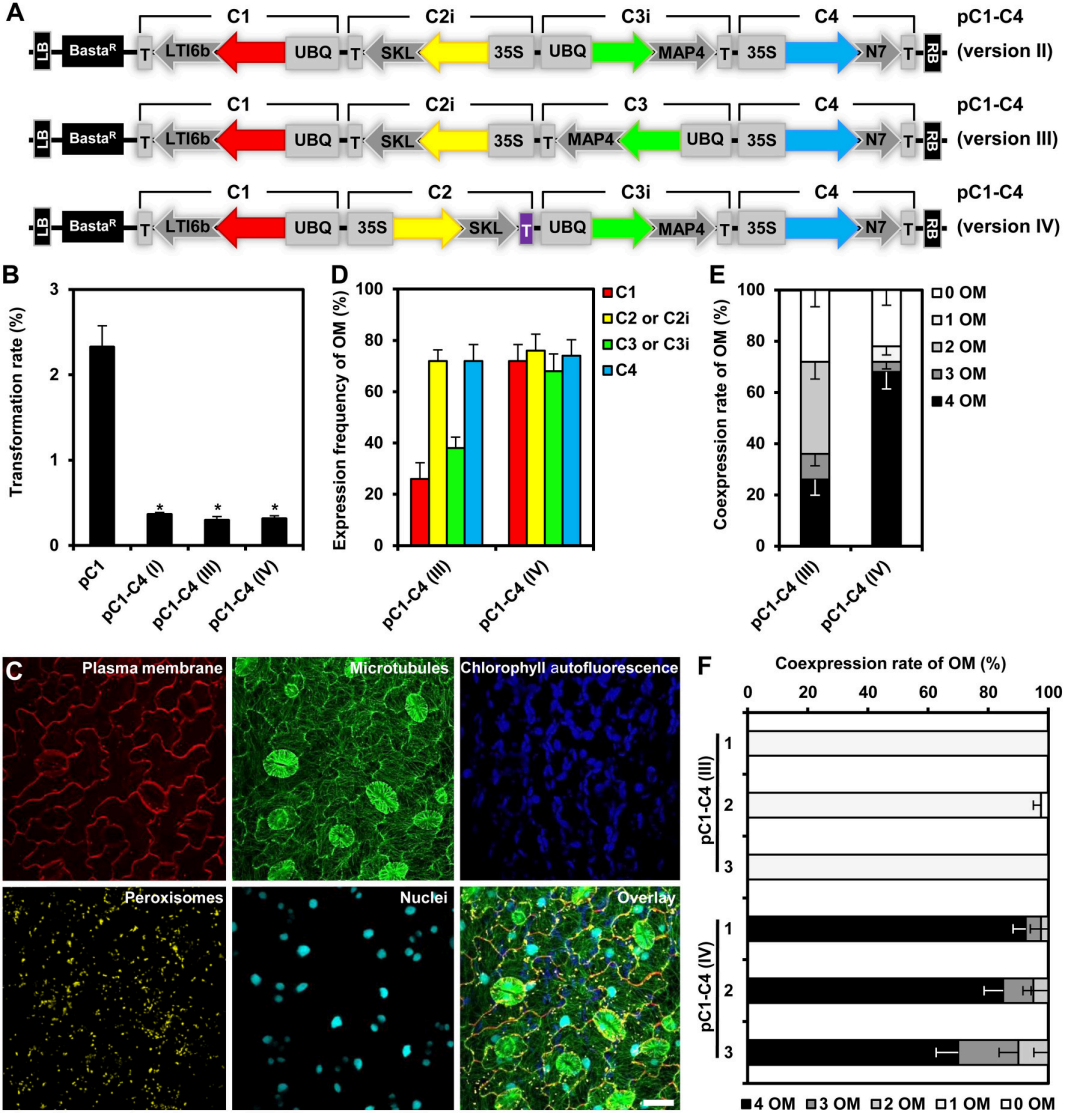
**Design, transformation rates and gene expression efficiency of the quadruple-gene assemblies in stable *Arabidopsis* transformants**. **(A)** Structural organization of the T-DNAs of different quadruple-gene assemblies; pC1–C4 (version II; top), pC1–C4 (version III; middle) and pC1–C4 (version IV; bottom). C1, C2, C2i, C3, C3i, and C4 indicate the gene cassettes modules compositing the multigene assemblies. The vector backbone was amplified from the plasmid pGreenII. UBQ10: polyubiquitin 10 promoter; 35S, cauliflower mosaic virus CaMV 35S promoter; T, terminator of the cauliflower mosaic virus 35S (gray), and terminator of octopine synthase (purple); red, yellow, green, and turquoise arrows: fluorescent protein encoding genes of mKate2, TagRFP-T, Venus and mTurquoise2, respectively; LTI6b: low temperature induced protein as a membrane marker; SKL: peroxisomes targeting sequence; MAP4: microtubule binding domain of the mouse microtubule-associated protein 4; N7: nuclear localization signal; Basta^R^: selectable marker confers Basta resistance in plants; Kan^R^: selectable marker confers kanamycin resistance in *E. coli* and *A. tumefaciens*; LB/RB: left/right borders of T-DNA. The figures are not drawn to scale. **(B)** Stable transformation rates of the multigene T-DNAs indicated in **(A)** calculated as the percent of the Basta-resistant *Arabidopsis* plants from T1 seeds. The data represent the means ± SEM of three experiments. Asterisks indicate significant differences in comparison to pC1 using student *t*-test, *p* < 0.01. pC1 contains a single gene cassette (UBQ10-mKate2-LTI6b-T35S) in addition to the Basta selectable marker. **(C)** Leaf cells of *Arabidopsis* stably expressing quadruple fluorescent organelle markers encoded from pC1–C4 (version IV). Chlorophyll autofluorescence is provided as a control. The images represent the maximum projection of z-stacks obtained by confocal laser scanning microscopy. Scale bar = 20 μm. **(D)** Expression frequency of organelle markers (OM) for C1 (membrane marker), C2 or C2i (peroxisomes marker), C3 or C3i (microtubules marker), or C4 (nuclear marker). Data represent percentages ± SEM (*n* = 50 plants). **(E)** Coexpression rate of organelle markers (OM) in T1 generation of transgenic plants harboring the T-DNAs indicated in **(A)**. *n* = 50 plants. **(F)** Coexpression rate of OMs in the T2 generation of three independent transgenic lines harboring the T-DNAs C1–C4 (version III) or C1–C4 (version IV). Please, note that offspring of T1 plants which coexpressed all four OMs was used. *n* = 40 plants.

We reasoned that one potential explanation for the reduced coexpression efficiency observed with our triple and quadruple assemblies may be the high homology between the assembled genes, encoding Kate2 and TagRFP-T, as well as Venus and mTurquoise2, which share 96 and 97% sequence similarity, respectively. Moreover, these homologous genes were organized in inverted orientations within our original assemblies (Figure 3E). It is well known that inverted repeats may enhance DNA methylation and consequently trigger gene silencing (Stam et al., 1998). In addition, gene silencing may also be enhanced because of a possible transcriptional read-through from the oppositely oriented gene cassettes C2 and C3 (Figure 3E). Consequently, we reconsidered and changed our assembly design strategy. To this end, we reversed the direction of the C2 and C3 cassettes by PCR amplification using oligonucleotide primers that swap the original *SfiI*-C and *SfiI*-D, and *SfiI*-E and *SfiI*-F sites (Figure 1A), respectively, to produce the inverted gene cassettes C2i and C3i, respectively (Figure 6A). C2i and C3i were ligated into the progenitor vector backbones of pC2 and pC3 using the *SfiI* cloning sites to generate the binary vectors pC2i and pC3i, respectively. In addition, we replaced the sequences for Tocs with T35S using the cloning sites *SpeI* and *SfiI*-C in pC2i to test whether the identity of the transcriptional terminator influences coexpression efficiency (Figure 6A). We used combinations of the *SfiI*-cleaved gene cassettes C1, C2, C2i, C3, C3i, and C4, as previously described for constructing pC1–C4 (version I), to produce three different vectors harboring quadruple-gene assemblies, which we named pC1–C4 (version II), pC1–C4 (version III), and pC1–C4 (version IV; Figure 6A). Subsequently, we transiently expressed these newly developed quadruple-gene assemblies in *N. benthamiana*, and used CLSM to detect the encoded fluorescent organelle markers. For the pC1–C4 (version II), we only detected expression of either one or three organelle markers in two independent experiments (Supplementary Figures S4A,B). Therefore, pC1–C4 (II) was excluded from subsequent stable gene expression analysis, as it failed to coexpress the four gene fusions. For pC1–C4 (version III) and pC1–C4 (version IV) all four organelle markers were repeatedly detectable by CLSM (Supplementary Figure S4A) and fully coexpressed in all investigated cells (Supplementary Figure S4B).

Next, we analyzed the efficiency of stable transformation and multigene expression of pC1–C4 (version III) and pC1–C4 (version IV) designs in *Arabidopsis*. Again, plants transformed with either new quadruple-gene assembly showed lower transformation rates (0.32 and 0.31%, respectively) in comparison to those transformed with pC1 (2.32%), but had a comparable rate (0.36%) to the original pC1–C4 (version I; Figure 6B). As before, we selected 50 stable transformants per assembly and analyzed the expression of the respective fluorescent organelle markers by CLSM. With both remaining new quadruple assemblies, all four organelle markers could be observed in individual transformants (Figure 6C). To quantitatively evaluate the expression performance of each multigene assembly, we calculated the expression frequency of each individual organelle marker and their coexpression rates as previously described. These analyses revealed that each of the four organelle markers was expressed at similar frequency (68–74%) in plants carrying C1–C4 (version IV), whereas their expression frequency highly varied (26–72%) in plants carrying C1–C4 (version III; Figure 6D). These results show that the expression frequencies of the organelle markers encoded by C1–C4 (version III) and C1–C4 (version IV) were significantly enhanced compared to the original assembly C1–C4 (version I; Figure 5C), albeit with distinct robustness. Notably, improved multigene expression appears to be associated with avoidance of the opposing transcriptional orientation of the C2 and C3 cassettes in the C1–C4 (version III) and C1–C4 (version IV) assemblies (Figure 6A), supporting our early assumption that the design of the multigene affects the outcome of gene expression. We also observed that the increase in the expression frequency of the individual organelle markers (Figure 6D) positively correlated with their coexpression rates (Figure 6E). Interestingly, the coexpression rate of the four organelle markers observed in plants carrying C1–C4 (version IV) was more than two-fold higher (68%) if compared to plants transformed with C1–C4 (version III), which showed a coexpression rate of 26% (Figure 6E). Apparently, these differences result mainly from suppressed expression of the organelle markers encoded by C1 and C3 in C1–C4 (version III; Figure 6D). The assemblies C1–C4 (version III) and C1–C4 (version IV) have similar modular organization with one gene showing outward orientation followed by three consecutive genes with the same directionality but opposite outward orientation (Figure 6A). However, the second gene cassette within the assemblies, C2i and C2 harbor different terminators T35S and Tocs, respectively (Figure 6A), suggesting that the outperformance of the multigene coexpression mediated by C1–C4 (version IV) is attributed to the Tocs terminator. Possible explanations may be that the Tocs sequence confers a stronger transcriptional termination or prevents transcriptional read-through. Alternatively, or additionally, the lower overall sequence homology within the multigene construct may also result in reduced gene silencing effects. Regardless of what the exact reason for the observed differences may be, our results corroborate the importance of assembly design for multigene expression. This conclusion is also supported by experiments in which we analyzed transgenerational stability of the organelle marker genes in the filial T2 generation. In these experiments we selected three independent transgenic lines of the T1 generation harboring the constructs C1–C4 (version III) and C1–C4 (version IV), which coexpressed all four organelle markers. Intriguingly, all T2 plants harboring the T-DNA of the C1–C4 (version III) expressed only one organelle marker, i.e., the peroxisomal marker encoded by C2i (Figure 6F; Supplementary Figure S5). In marked contrast, most (i.e., >70%) of the T2 offspring deriving from the C1–C4 (version IV) transgenic plants coexpressed all four organelle markers (Figure 6F; Supplementary Figure S5). Interestingly, only the UBQ10 promoter-driven organelle markers C1 and C3i showed reduced transgenerational stability, whereas 35S promoter-driven markers C2 and C4 could be detected in all T2 plants (Supplementary Figure S5). Together, these findings also highlight the importance of the 5′ regulatory sequences for transgenerational expression and thus, multigene assembly design. Accordingly, we recommend utilization of the C1–C4 (version IV) design as the optimal COLORFUL-Circuit assembly for coexpression of up to four genes.

### COLORFUL-circuit allows spatio-temporal visualization of multiple organelle dynamics during biotrophic plant-microbe interactions

**Figure 7:**
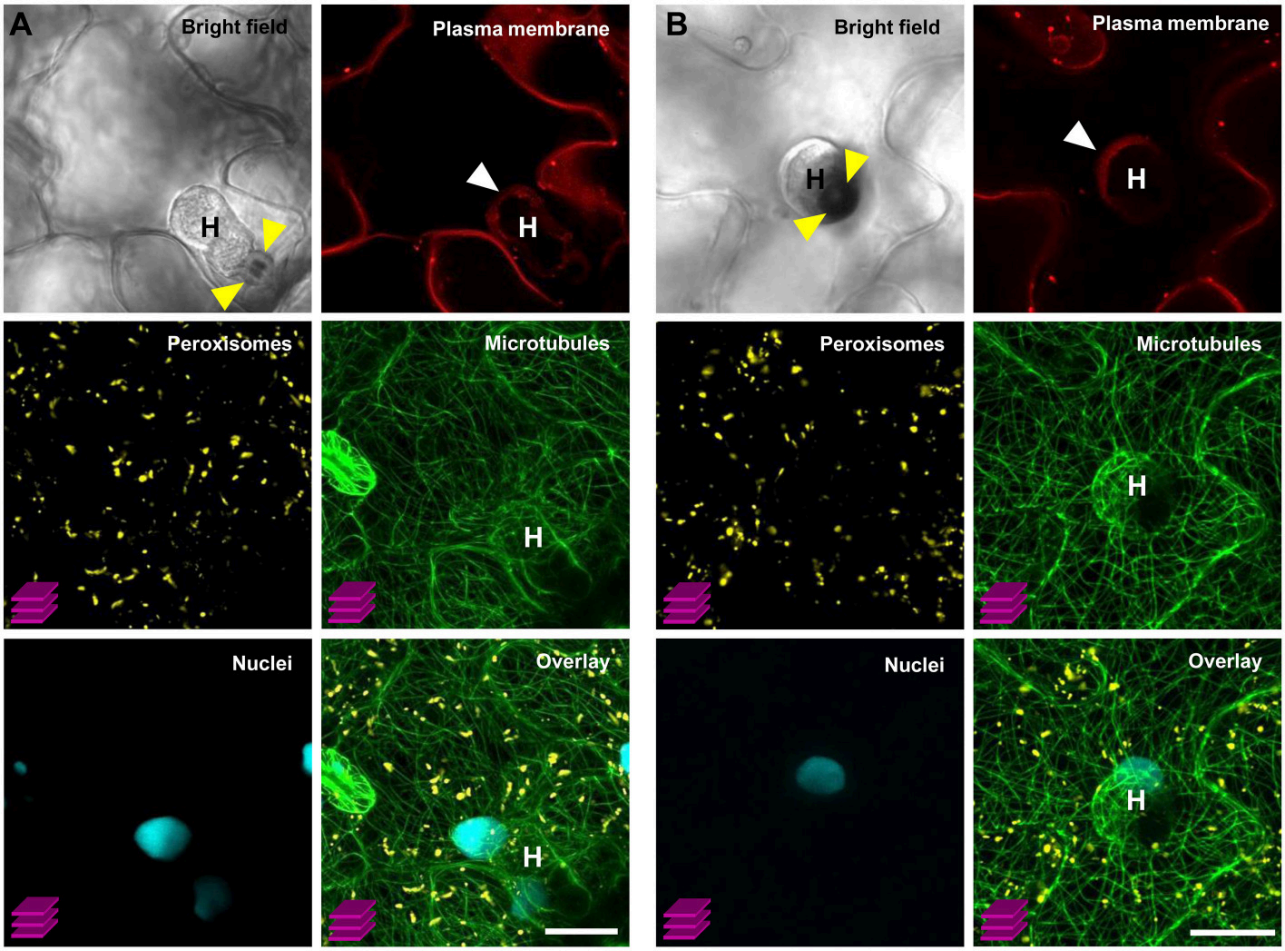
**Simultaneous visualization of multiple subcellular markers at mature haustorial complexes of *G. orontii* in *Arabidopsis***. Conidiospores of *G. orontii* were used to inoculate 4-week old *Arabidopsis* plants expressing the multiorganelle markers C1–C4 (version IV) from Figure 6A. Sites of fungal attack were analyzed at 4 days post inoculation by CLSM (z-stacks, except for the images of bright field and plasma membrane marker). **(A)** Side-view and **(B)** top-view of distinct mature haustoria (marked with a white H) within epidermal leaf cells. Callose encasement of the haustorial neck in **(A)** and the callose papilla in **(B)** are indicated with yellow arrowheads. Haustoria are surrounded by the extrahaustorial membrane (white arrowhead). Peroxisomes are homogenously distributed within the cell. Nuclei are located in close proximity to the haustorial complexes. Microtubules envelop the haustorial complexes. Scale bar = 20 μm.

As an exemplary case study, we tested the utility of our multigene expressing line C1–C4 (version IV) by simultaneously monitoring the subcellular behavior of organelles that occur during the compatible interaction between Arabidopsis and the obligate biotrophic powdery mildew fungus *G. orontii*. In general, upon contact with the leaf surface of a potential host plant the conidiospores of powdery mildew fungi germinate and develop penetration organs called appressoria, which they use to invade through the host outer epidermal cuticle and cell wall. Subsequently, in compatible interactions, haustorial complexes are established within penetrated epidermal cells, and serve the uptake of plant nutrients and the transfer of fungal effector molecules (Koh et al., 2005; Micali et al., 2008, 2011). Typically, haustoria invaginate and modify the host plant plasma membrane to form a specialized exchange interface with the host cytoplasm called extrahaustorial membrane (EHM; Koh et al., 2005; Micali et al., 2011). Nutrient uptake results in development of secondary hyphae and radial ectoparasitic colonization of the host that is accompanied by secondary haustoria formation in neighboring epidermal cells (Micali et al., 2008). Finally, an epiphytic network of fungal hyphae covers the leaf surface and new conidiospores are produced, giving rise to the typical powdery mildew disease symptoms (Micali et al., 2008). Previous studies which focused on early compatible and incompatible Arabidopsis–powdery mildew interactions revealed that attempted invasion and successful host colonization impact dramatically on plant cell membrane integrity, cytoskeleton structure and organelle dynamics (Koh et al., 2005; Takemoto et al., 2006; Chandran et al., 2010; Micali et al., 2011; Hardham, 2013), thus reflecting either efficient cell-autonomous defense mechanisms or the establishment of compatibility. However, studies aiming at simultaneous observation of multiple FP-tagged membranes, structures and organelles during compatible and incompatible Arabidopsis–powdery mildew interactions have not yet been conducted. Here, we used the COLORFUL-Circuit line C1–C4 (version IV) which coexpresses FP-tagged reporter constructs for the plasma membrane, peroxisomes, microtubules and the nucleus to simultaneously analyze the subcellular behavior of these organelles at compatible Arabidopsis–*G. orontii* interaction sites with a focus on mature haustoria. To this end, we inoculated 4-week old transgenic Arabidopsis Col-0 plants harboring C1–C4 (version IV) with conidiospores of *G. orontii* and used CLSM to visualize the localization of the respective reporter constructs at sites of established haustoria at 4 days post inoculation. The representative images shown in Figure 7 show two distinct mature haustoria in a side-view (Figure 7A) and a top-view perspective (Figure 7B), respectively. Both sites show that the plasma membrane marker LTI6b localizes to the EHM. This is interesting as former analyses performed with the same protein fused to GFP did not allow detection in the EHM of early haustoria induced by the compatible powdery mildew *Golovinomyces cichoracearum* (Koh et al., 2005). This may suggest that distinct powdery mildew species induce specific EHMs that differ in regard to protein composition. An alternative explanation, which we consider more likely, is that the protein repertoires recruited into the EHM vary in a developmentally dependent manner. Similarly, Micali et al. (2011) observed that the Arabidopsis resistance protein RPW8.2 accumulated at the EHM of mature haustoria but not at EHM of young haustoria. Interestingly, peroxisomes were homogenously distributed and did not accumulate close to the haustorial complexes as observed before by Koh et al. (2005) for early interaction sites with *G. cichoracearum*. Again, this may indicate development-dependent alterations in the subcellular behavior and localization dynamics of the individual reporter proteins. Future analysis should address this question with time-course CLSM experiments. Consistent with the previous analysis by Koh et al. (2005) we found a tight spatial association of the nuclei of the invaded epidermal plant cells with the haustorial complexes. Moreover, we found that microtubules envelop the mature haustorial complexes, supporting earlier observations by Takemoto et al. (2006) and Hardham (2013) that microtubules are subject to pathogen-induced structural reorganization. It is important to mention that the COLORFUL-Circuit system allows the easy and straightforward exchange of individual modules. Thus, it will be possible to simultaneously visualize for example the interplay of the actin and microtubule cytoskeleton for organellar transport and localized arrest, by exchanging the plasma membrane marker with an actin-filament associated protein, for example the actin-binding domain 2 of Arabidopsis fimbrin 1 (Wang et al., 2008). Moreover, other organelle markers such as those described by Nelson et al. (2007) can be used to generate transgenic plants for the purpose of parallel subcellular localization studies of up to four distinct compartments. It is important to notice, however, that our system does not only allow cell biological studies, like those described so far. Also, our versatile system offers the possibility to manipulate metabolite fluxes or even to introduce entirely novel biosynthetic pathways by simultaneously or spatio-temporally regulating production of respective enzymatically active proteins under control of adequate tissue- or stimulus-specific promoter sequences.

Taken together, we established a new vector system called “COLORFUL-Circuit” that allows multigene assembly, delivery, and *in planta* coexpression. The vectors can be flexibly customized by exchanging any module of the existing gene cassettes. COLORFUL-Circuit is privileged by the utilization of a single and inexpensive rare-cutting restriction enzyme, *SfiI*, which simplifies the assembly of large genomic DNA fragments. The usage of COLORFUL-Circuit assembly saves time for transient and stable multigene coexpression, and consequently provides biotechnological advantages for simultaneous improvement of multiple plant treats and engineering of complex metabolic pathways. Moreover, our system can be exploited for basic research questions, e.g., by utilization of fluorescent protein-tagged reporters. The future of plant cell biology and biotechnology is COLORFUL.

## Author contributions

HG and VL designed the experiments, HG and SL performed the experiments, HG, SL and VL analyzed and discussed the results and HG and VL wrote the paper.

### Conflict of interest statement

The authors declare that the research was conducted in the absence of any commercial or financial relationships that could be construed as a potential conflict of interest.
