# Supplementary material for: COLORFUL-Circuit: A Platform for Rapid Multigene Assembly, Delivery, and Expression in Plants
Source: Front Plant Sci. 2016 Mar 1;7:246. doi: 10.3389/fpls.2016.00246 (PMC4772762; doi:10.3389/fpls.2016.00246)
Supplement: Supplementary file 1 [file Table1.PDF]

Supplementary Table S1. Oligonucleotide sequences used for PCR amplification

Primer name	Primer Sequence	Purpose
oHG001	*RsrII*<br>ATCTACCATGGGGCCTCCCTGGCC**CGGTCCG**CGTGTCGACGAGTCAGTAATAAACG	F primer for UBQ10 and C1 casstte
oHG002	*BamHI* *RsrII*<br>GGCGA**GGATCC*****CGGACCG***CTGTTAATCAGAAAAACTCAG	R primer for UBQ10
oHG011	*BamHI*<br>TAGCT**GGATCC**ATGGTGAGCGAGCTGATTAAGG	F primer for mKate2
oHG035	*EcoRI*<br>TTAT**GAATTC**GGCCGCTGCCGCAGC	R primer for mKate2
oHG085	*EcoRI*<br>GGCC**GAATTC**AAGCGCTTGAAAATGAG	F primer for LTI6b
oHG051	*SpeI*<br>CTAG**ACTAGT**CGGGGAAATTCGAGCTCTCAAAAGGTGATG	R primer for LTI6b
oHG021	*SpeI*<br>GGCCG**ACTAGT**CTAGAGTCCGCAAAAATCACC	F primer for T35S
oHG010	*SfiI*<br>ATATTGGCGCGCC**GGCCTCCGTGGCC**GGTCACTGGATTTTGG	R primer for T35S
oHG007	*SfiI*<br>ATATTCCATGG**GGCCTGGTTGGCC**CGGTCCGCGTGTC	F primer for C1.1 cassette
oHG008	*SfiI*<br>ATATTGGCGCGCC**GGCCTCTGTGGCC**GGTCACTGGATTTTGG	R primer for C1.1 cassette
oHG005	*SfiI*<br>ATATTCCATGG**GGCCTGGGTGGCC**CGGTCCGCGTGTC	F primer for C1.2 cassette

oHG006	*SfiI*<br>ATATTGGCGCGCC**GGCCTCAGTGGCC**GGTCACTGGATTTTGG	R primer for C1.2 cassette
oHG045	*SfiI*<br>ATATT**GGCCACCCTGGCC**CGGTCCGCGTGTCG	F primer for C1.3 cassette
oHG046	*SfiI*<br>ATATT**GGCCAGACTGGCC**GGTCACTGGATTTTGG	R primer for C1.3 cassette
oHG003	*SfiI*<br>ATCTA**GGCCTCGGTGGCC**GGCGCCCCAAACCGAAGGCGGGAAAC	F primer for C1 vector backbone
oHG031	*SfiI*<br>ATCTA**GGCCAGGTAGGCC**GGGATCAGATTGTCGTTTCC	R primer for C1 vector backbone
oHG033	*SfiI*<br>ATCTA**GGCCAACCAGGCC**GGCGCCCCAAACCGAAGGCGGGAAAC	F primer for C1.1 vector backbone
oHG034	*SfiI*<br>ATATA**GGCCACAGAGGCC**GGGATCAGATTGTCGTTTCC	R primer for C1.1 vector backbone
oHG032	*SfiI*<br>ATCTA**GGCCACTGAGGCC**GGCGCCCCAAACCGAAGGCGGGAAAC	F primer for C1.2 vector backbone
oHG028	*SfiI*<br>ATCTA**GGCCTCCCTGGCC**GGGATCAGATTGTCGTTTCC	R primer for C1.2 vector backbone
oHG017	*SfiI*<br>ATCTA**GGCCTGGGTGGCC**GGCGCCCCAAACCGAAGGCGGGAAAC	F primer for C1.3 vector backbone
oHG004	*SfiI*<br>ATCTA**GGCCTGTCTGGCC**GGGATCAGATTGTCGTTTCC	R primer for C1.3 vector backbone

oHG124	*BamHI*<br>CGGTCCG**GGATCC**ATGGTGTCTAAGGGCGAAG	F primer for TagRFP-T-SKL
oHG123	*SpeI*<br>TAGCT**ACTAGT**TTACAGCTTCGATCTTCCGGACTTGTACAGCTCG	R primer for TagRFP-T-SKL
oHG019	*BamHI*<br>TAGCT**GGATCC**TGTATGGTGAGCAAGGGCGAGGAGC	F primer for Venus and mTurquoise2
oHG036	*EcoRI*<br>TAGC**GAATTC**GCAATTCCGGACTTGTACAGCTCG	R primer for Venus and mTurquoise2
oHG092	*Overlapping sequence for Gibson Assembly*<br>**ACAAGCACGCGGTGATGTCC**CGGCAAGAAGAAGCAAAG	F primer for MAP4
oHG093	*Overlapping sequence for Gibson Assembly*<br>**GGTGATTTTTGCGGACTCTA**GACTAGTTTAACCTCCTGCAGGAAAGTGGC	R primer for MAP4
oHG030	*Overlapping sequence for Gibson Assembly*<br>**CGAGCTGTACAAGTCCGGAA**TTGGCGAATTCAAGCGTGAAG	F primer for N7
oHG020	*Overlapping sequence for Gibson Assembly*<br>**GGTGATTTTTGCGGACTCTA**GATTACTCTTCTTCTTGATCAGC	R primer for N7
oHG125	*BamHI* *RsrII*<br>CACCAT**GGATCC*CGGACCG***CGGTCCTCTCCAAATGAAATG	Amplification of 35S
oHG126	*RsrII*<br>GGCCTGGTTGGC**CCGGTCCG**GTCCCCAGATTAGCCTTTTC	Amplification of 35S
oHG128	*SfiI*<br>ACAATCTGATCCC**GGCCTCTGTGGCC**GTTTGTTATTGTGGCGCTC	Amplification of Toct
oHG137	*SpeI*<br>GAAGATCGAAGCTGTAA**ACTAGT**CCACCCCCTGCTTTAAT	Amplification of Toct

	GAG	
oHG043	*SfiI*<br>ATATT**GGCCTCTGTGGCC**CGGTCCGCGTGTCG	F primer for C2i cassette
oHG044	*SfiI*<br>ATATT**GGCCTGGTTGGCC**GGTCACTGGATTTTGG	R primer for C2i cassette
oHG134	*SfiI*<br>ATATA**GGCCACAGAGGCC**CGGTCCGCGTGTCGAC	F primer for C3i cassette
oHG135	*SfiI*<br>TATATT**GGCCAGGGTGGCC**GGTCACTGGATTTTGG	R primer for C3i cassette
oHG120	*SfiI*<br>ATCTA**GGCCTACCTGGCC**CGGTACCCAATTCGCCCTATAG	F primer for pC2-C3 vector backbone
oHG118	*SfiI*<br>ATCTA**GGCCTCCCTGGCC**CCAAAATCCAGTGACCGGGC	R primer for pC2-C3 vector backbone
oHG112	*SfiI*<br>ATCTA**GGCCTCGGTGGCC**CGGTACCCAATTCGCCCTATAG	F primer for pC1-C3 vector backbone
oHG118	*SfiI*<br>ATCTA**GGCCTCCCTGGCC**CCAAAATCCAGTGACCGGGC	R primer for pC1-C3 vector backbone
oHG112	*SfiI*<br>ATCTA**GGCCTCGGTGGCC**CGGTACCCAATTCGCCCTATAG	F primer for pC1-C4 vector backbone
oHG113	*SfiI*<br>ATCTA**GGCCTGTCTGGCC**CCAAAATCCAGTGACCGGGC	R primer for pC1-C4 vector backbone
