# Supplementary material for: COLORFUL-Circuit: A Platform for Rapid Multigene Assembly, Delivery, and Expression in Plants
Source: Front Plant Sci. 2016 Mar 1;7:246. doi: 10.3389/fpls.2016.00246 (PMC4772762; doi:10.3389/fpls.2016.00246)
Supplement: Supplementary file 2 [file Table2.PDF]

Supplementary Table S2. Cleavage site frequency of the restriction enzymes *SfiI*, *BsmBI*, *BsaI* and *SapI* occurring in individual chromosomes of *A. thaliana* (ecotype Col-0)

**Name of chromosome**	**Size (MB*)**	**Number of cleavage sites**			
		SfiI	***BsaI***	***BsmBI***	***SapI***
Chromosome 1	30.427671	91	7945	8298	4585
Chromosome 2	19.698289	66	5188	5461	2986
Chromosome 3	23.45983	72	6394	6598	3542
Chromosome 4	18.585056	51	4953	5138	2774
Chromosome 5	26.975502	91	7198	7367	4052
**Total**	119.146348	371	31678	32862	17939
**Number of cleavage sites per MB***		3.1	265.9	275.9	150.6

*megabase
