# Supplementary material for: COLORFUL-Circuit: A Platform for Rapid Multigene Assembly, Delivery, and Expression in Plants
Source: Front Plant Sci. 2016 Mar 1;7:246. doi: 10.3389/fpls.2016.00246 (PMC4772762; doi:10.3389/fpls.2016.00246)
Supplement: Supplementary file 3 [file Table3.PDF]

Supplementary Table S3. Cleavage site frequency of the restriction enzymes *SfiI*, *BsmBI*, *BsaI* and *SapI* occurring in individual chromosomes of rapeseed (*Brassica napus* cultivar Darmor-*bzh*)

Name of chromosome	Size (MB*)	Number of cleavage sites			
		SfiI	*BsaI*	*BsmBI*	*SapI*
Chromosome An1	23.267856	88	5193	6052	2704
Chromosome An2	24.793737	113	5698	6036	2839
Chromosome An3	29.767490	102	7107	8157	3792
Chromosome An4	19.151660	61	4442	4579	2126
Chromosome An5	23.067598	84	5129	5993	2843
Chromosome An6	24.396386	90	5988	6487	3125
Chromosome An7	24.006521	90	5473	6151	2792
Chromosome An8	18.961941	78	4532	4912	2408
Chromosome An9	33.865340	107	7692	8602	3891
Chromosome An10	17.398227	78	4344	4923	2380
Chromosome Cn1	38.829317	216	9325	10316	4424
Chromosome Cn2	46.221804	285	11138	11474	5105
Chromosome Cn3	60.573394	294	14529	15562	6554
Chromosome Cn4	48.930237	290	11294	12128	5279
Chromosome Cn5	48.185227	260	10679	11715	5292
Chromosome Cn6	37.225952	187	9252	9695	4456
Chromosome Cn7	44.770477	229	10864	11528	5123

Chromosome Cn8	38.477087	231	9870	10589	4676
Chromosome Cn9	48.508220	307	12021	12453	5572
**Total**	650.398471	3190	154570	167352	75381
**Number of cleavage sites per MB***		4.9	237.7	257.3	115.9

*megabase, chromosome sizes without the randomly assembled nucleotide sequences
