# Supplementary material for: COLORFUL-Circuit: A Platform for Rapid Multigene Assembly, Delivery, and Expression in Plants
Source: Front Plant Sci. 2016 Mar 1;7:246. doi: 10.3389/fpls.2016.00246 (PMC4772762; doi:10.3389/fpls.2016.00246)
Supplement: Supplementary file 4 [file Table4.PDF]

Supplementary Table S4. Cleavage site frequency of the restriction enzymes *SfiI*, *BsmBI*, *BsaI* and *SapI* occurring in individual chromosomes of tomato (*Solanum lycopersicum* cultivar Heinz 1706)

**Name of chromosome**	**Size (MB*)**	**Number of cleavage sites**			
		SfiI	***BsaI***	***BsmBI***	***SapI***
Chromosome 1	98.543444	523	19507	9297	6899
Chromosome 2	55.340444	327	10430	5450	3916
Chromosome 3	70.787664	265	14528	6810	4931
Chromosome 4	66.470942	315	14075	6536	4655
Chromosome 5	65.875088	276	14950	6486	4472
Chromosome 6	49.751636	249	9661	4975	3469
Chromosome 7	68.045021	324	15847	6600	4702
Chromosome 8	65.866657	233	14555	6274	4565
Chromosome 9	72.482091	295	16439	6858	4930
Chromosome 10	65.527505	261	14805	6594	4642
Chromosome 11	56.302525	263	11974	5790	3857
Chromosome 12	67.145203	328	15199	6569	4749
**Total**	703.594776	3136	152463	68942	48888
**Number of cleavage sites per MB***		4.5	216.7	98	69.5

*megabase
