# Supplementary material for: COLORFUL-Circuit: A Platform for Rapid Multigene Assembly, Delivery, and Expression in Plants
Source: Front Plant Sci. 2016 Mar 1;7:246. doi: 10.3389/fpls.2016.00246 (PMC4772762; doi:10.3389/fpls.2016.00246)
Supplement: Supplementary file 5 [file Table5.pdf]

Supplementary Table S5. Cleavage site frequency of the restriction enzymes *SfiI, BsmBI, BsaI* and *SapI* occurring in individual chromosomes of rice (*Oryza sativa* cultivar Nipponbare)

**Name of chromosome**	**Size (MB*)**	**Number of cleavage sites**			
		SfiI	***BsaI***	***BsmBI***	***SapI***
Chromosome 1	43.270923	1863	11940	15811	5973
Chromosome 2	35.937250	1303	9745	12657	4789
Chromosome 3	36.413819	1400	10053	13406	4992
Chromosome 4	35.502694	1648	9621	12445	4879
Chromosome 5	29.958434	1292	8079	10476	4200
Chromosome 6	31.248787	1325	8120	10669	4255
Chromosome 7	29.697621	1172	7836	9928	3859
Chromosome 8	28.443022	1189	7651	9382	3818
Chromosome 9	23.012720	978	6120	8004	3036
Chromosome 10	23.207287	950	6190	7809	3034
Chromosome 11	29.021106	1123	7544	9250	3723
Chromosome 12	27.531856	1084	7206	8786	3685
**Total**	373.245519	13464	88165	112812	44270
**Number of cleavage sites per MB***		40.8	267.2	341.9	134.2

*megabase
