# Supplementary material for: COLORFUL-Circuit: A Platform for Rapid Multigene Assembly, Delivery, and Expression in Plants
Source: Front Plant Sci. 2016 Mar 1;7:246. doi: 10.3389/fpls.2016.00246 (PMC4772762; doi:10.3389/fpls.2016.00246)
Supplement: Supplementary file 6 [file Image1.PDF]

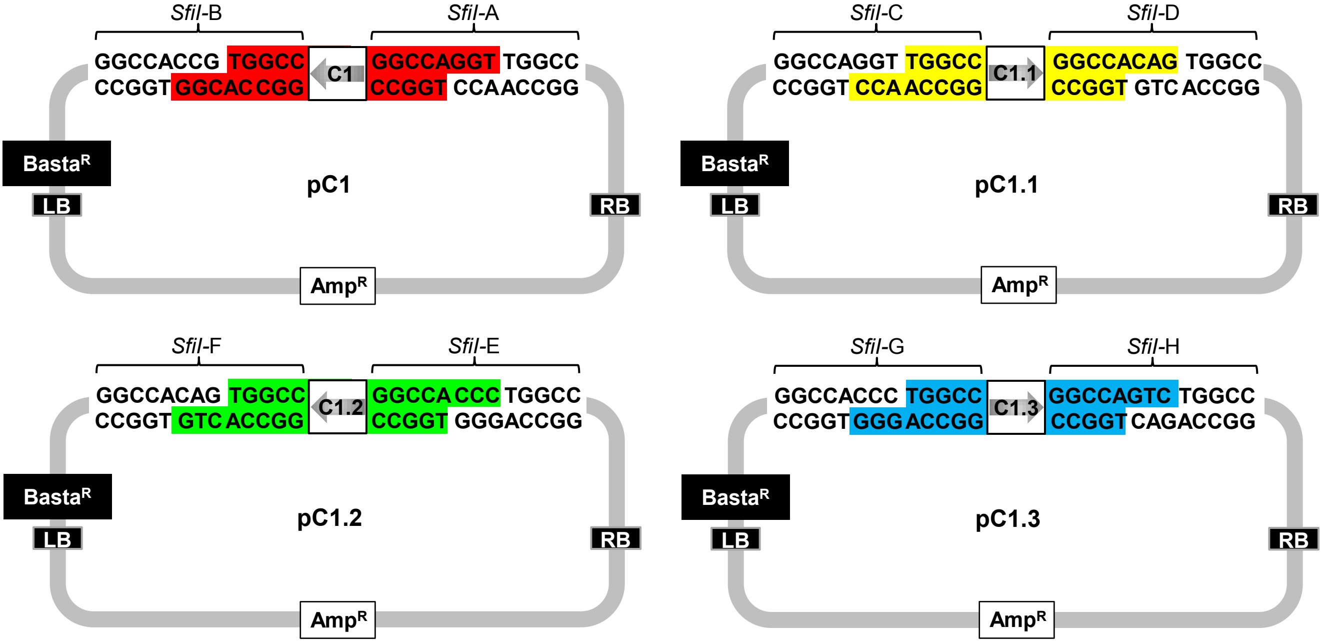

Supplementary Figure S1. Schematic representation of the binary vectors hosting the gene cassettes C1, C1.1, C1.2 and C1.3*.* The binary vector backbones were generated to harbor terminal *SfiI* overhangs compatible with the *SfiI* overhangs flanking the corresponding gene cassette C1 (1896 bp), C1.1 (1896 bp), C1.2 (1896 bp) or C1.3 (1896 bp) to produce the binary vectors pC1, pC1.1, pC1.2, pC1.3, respectively. Basta^R: selectable marker conferring Basta resistance in plants; Amp^R: selectable marker conferring ampicillin resistance in *E. coli* and *A. tumefaciens*; LB/RB: left/right borders of T-DNA. *SfiI*-A, *SfiI*-B, *SfiI*-C, *SfiI*-D, *SfiI*-E, *SfiI*-F, *SfiI*-G and *SfiI*-H are unique *SfiI* restriction sequences that flank the gene cassettes C1, C1.1, C1.2 and C1.3 as depicted in the figure. Colors denote the *SfiI* overhangs belonging to the same gene cassette. The gray arrows indicate transcriptional orientations of the gene cassettes. The figure is not drawn to scale.
