# Supplementary material for: COLORFUL-Circuit: A Platform for Rapid Multigene Assembly, Delivery, and Expression in Plants
Source: Front Plant Sci. 2016 Mar 1;7:246. doi: 10.3389/fpls.2016.00246 (PMC4772762; doi:10.3389/fpls.2016.00246)
Supplement: Supplementary file 7 [file Image2.PDF]

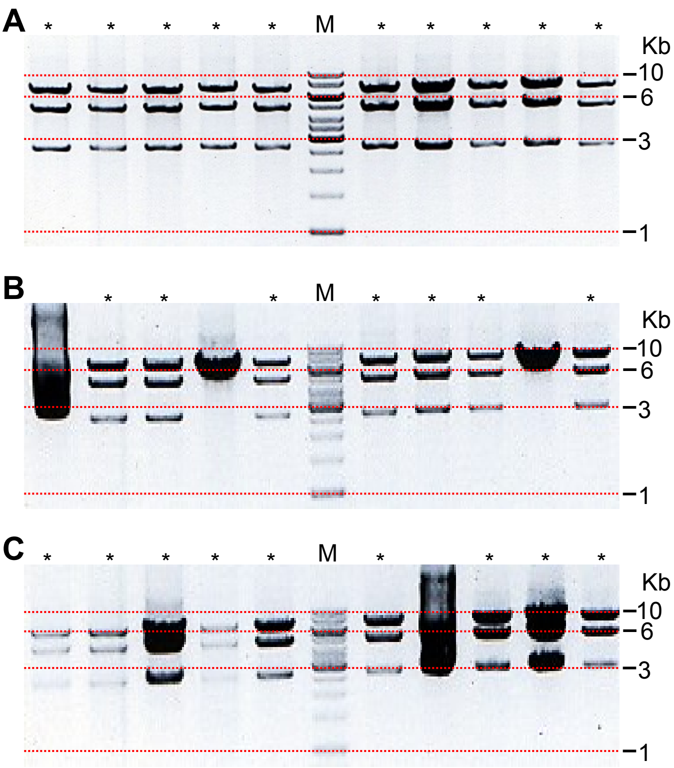

Supplementary Figure S2. *SalI* digestion of the14.2-Kb plasmid pC1-C4 (version I) isolated from 10 different *E. coli* clones.* indicate correct plasmids displaying three DNA fragments; 2.7, 4.8 and 6.7 Kb. Red discontinuous lines cross GeneRuler™ 1-Kb DNA ladder (M) at the indicated DNA fragment sizes. A, B and C each represents result obtained from independent experiment.
