# Supplementary material for: COLORFUL-Circuit: A Platform for Rapid Multigene Assembly, Delivery, and Expression in Plants
Source: Front Plant Sci. 2016 Mar 1;7:246. doi: 10.3389/fpls.2016.00246 (PMC4772762; doi:10.3389/fpls.2016.00246)
Supplement: Supplementary file 8 [file Image3.PDF]

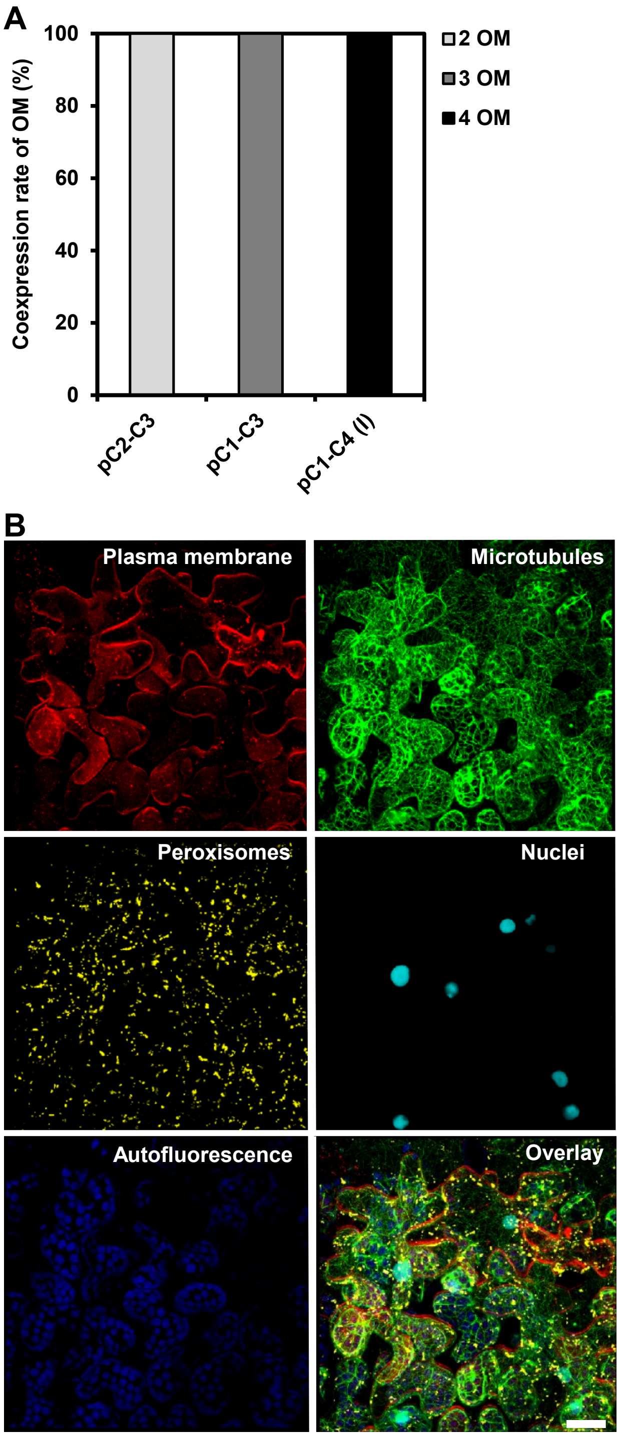

Supplementary Figure S3. Efficiency of transient gene expression obtained from COLORFUL-Circuits assemblies pC2-C3, pC1-C3 and pC1-C4 (version I) in agroinfiltrated leaves of *N. benthamiana*. **(A)** Coexpression rate of organelle markers (OM) calculated as percent of agroinfiltrated cells that coexpress different numbers of organelle markers. n = 100 cells for each construct. **(B)** Leaf cells of *N. benthamiana* infiltrated with *A. tumefaciens* harboring the quadruple-gene assembly pC1-C4 (version I) showing the expression of the four organelle markers encoded from the gene cassettes C1 (membrane marker), C2 or C2i (peroxisomes marker), C3, C3i (microtubules marker) or C4 (nuclear marker). Chlorophyll autofluorescence provided as a control. The images represent the maximum projection of z-stacks obtained by confocal laser scanning microscopy. Scale bar = 30 μm.
