# Supplementary material for: COLORFUL-Circuit: A Platform for Rapid Multigene Assembly, Delivery, and Expression in Plants
Source: Front Plant Sci. 2016 Mar 1;7:246. doi: 10.3389/fpls.2016.00246 (PMC4772762; doi:10.3389/fpls.2016.00246)
Supplement: Supplementary file 9 [file Image4.PDF]

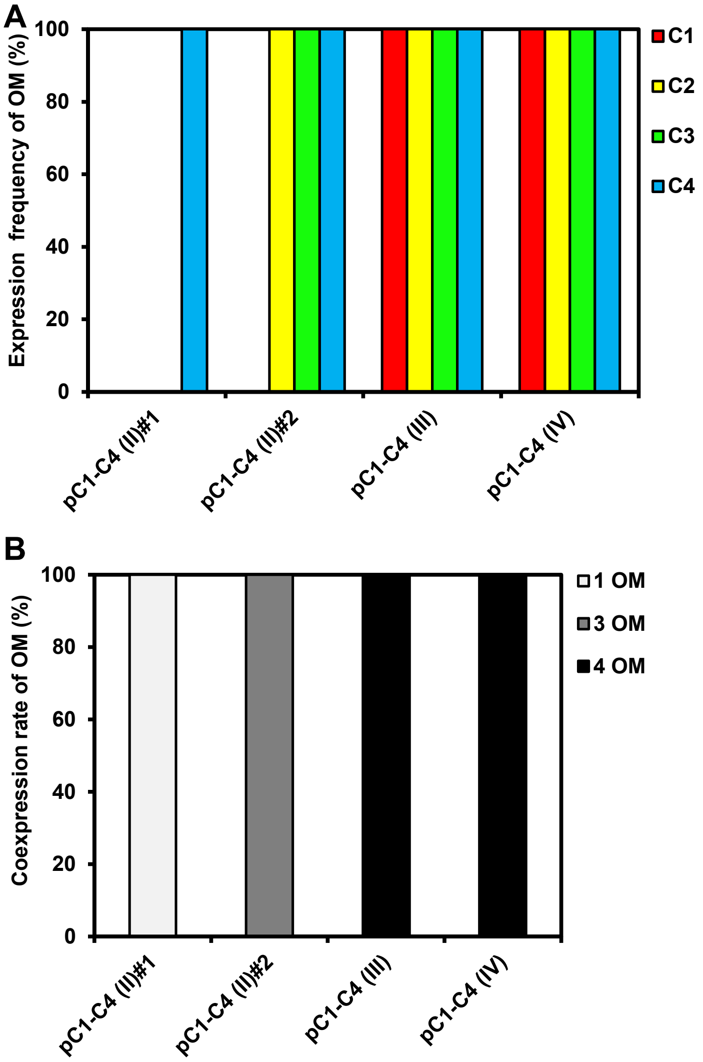

Supplementary Figure S4. Efficiency of transient gene expression encoded from the quadruple-gene assemblies pC1-C4 (version II), pC1-C4 (version III) and pC1-C4 (version IV) in agroinfiltrated leaves of *N. benthamiana*. **(A)** Expression frequency of organelle markers (OM) calculated as percent of agroinfiltrated cells that express OM encoded from the gene cassette C1 (membrane marker), C2 (peroxisomes marker), C3 (microtubules marker) or C4 (nuclear marker). n = 100 cells for each construct. **(B)** Coexpression rate of organelle markers (OM) calculated as percent of agroinfiltrated cells that coexpress different numbers of organelle markers. n = 100 cells for each construct.
