# Supplementary material for: COLORFUL-Circuit: A Platform for Rapid Multigene Assembly, Delivery, and Expression in Plants
Source: Front Plant Sci. 2016 Mar 1;7:246. doi: 10.3389/fpls.2016.00246 (PMC4772762; doi:10.3389/fpls.2016.00246)
Supplement: Supplementary file 10 [file Image5.PDF]

A

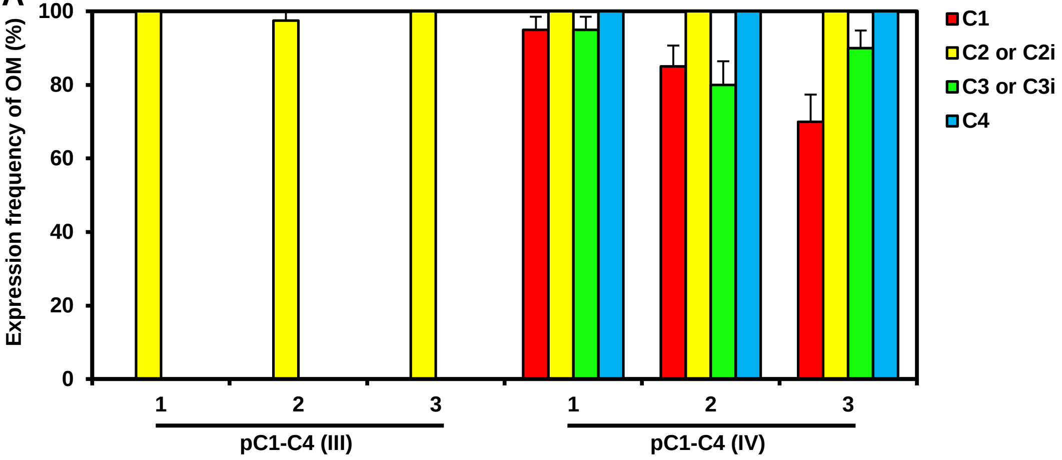

Supplementary Figure S5. Expression frequency of organelle markers (OM) for C1 (membrane marker), C2 or C2i (peroxisomes marker), C3 or C3i (microtubules marker) or C4 (nuclear marker) in the T2 generation of three independent transgenic lines harboring the T-DNAs C1-C4 (version III) or C1-C4 (version IV). Data represent percentages ± SEM (n = 40 plants).
